# Increased sporulation underpins adaptation of *Clostridium difficile* strain 630 to a biologically–relevant faecal environment, with implications for pathogenicity

**DOI:** 10.1038/s41598-018-35050-x

**Published:** 2018-11-12

**Authors:** Nigel George Ternan, Nicola Diana Moore, Deborah Smyth, Gordon James McDougall, James William Allwood, Susan Verrall, Christopher Ian Richard Gill, James Stephen Gerard Dooley, Geoff McMullan

**Affiliations:** 1 Nutrition Innovation Centre for Food and Health (NICHE), School of Biomedical Sciences, University of Ulster, Coleraine, Co. Londonderry, N. Ireland BT52 1SA United Kingdom; 2 Environmental and Biochemical Sciences Group, The James Hutton Institute, Invergowrie, Dundee, Scotland DD2 5DA United Kingdom; 3 Institute for Global Food Security, School of Biological Sciences, Medical Biology Centre, Queen’s University, Belfast, Northern Ireland BT9 7BL United Kingdom

## Abstract

*Clostridium difficile* virulence is driven primarily by the processes of toxinogenesis and sporulation, however many *in vitro* experimental systems for studying *C. difficile* physiology have arguably limited relevance to the human colonic environment. We therefore created a more physiologically–relevant model of the colonic milieu to study gut pathogen biology, incorporating human faecal water (FW) into growth media and assessing the physiological effects of this on *C. difficile* strain 630. We identified a novel set of *C. difficile*–derived metabolites in culture supernatants, including hexanoyl– and pentanoyl–amino acid derivatives by LC-MS^n^. Growth of *C. difficile* strain 630 in FW media resulted in increased cell length without altering growth rate and RNA sequencing identified 889 transcripts as differentially expressed (p < 0.001). Significantly, up to 300–fold increases in the expression of sporulation–associated genes were observed in FW media–grown cells, along with reductions in motility and toxin genes’ expression. Moreover, the expression of classical stress–response genes did not change, showing that *C. difficile* is well–adapted to this faecal milieu. Using our novel approach we have shown that interaction with FW causes fundamental changes in *C. difficile* biology that will lead to increased disease transmissibility.

## Introduction

The Gram–positive spore–forming anaerobe *Clostridium difficile* is recognised as one of the major causes of health-care associated infections^1,2^ and exerts a negative and well–publicised impact on hospital morbidity and mortality rates^1^. *C. difficile* infection (CDI) can develop when broad spectrum antibiotics are deployed to treat underlying infections: they disrupt the body’s natural colonic microbiota thus allowing development of CDI if spores or cells of this multidrug–resistant pathogen are also present^3^. Colonisation of the host gastrointestinal tract depends on the germination of *C. difficile* spores, with subsequent growth of vegetative cells and the release of two large clostridial glycosylating toxins, toxin A and toxin B^4^. These toxins are responsible for the inflammation and epithelial tissue damage that results in rapid loss of fluid and consequent diarrhoea^5^. Clinical manifestations and severity of CDI vary from mild self-limiting diarrhoea to life-threatening pseudomembranous colitis and, in severe cases, death^6^.

While it is well established that gut microbiome disruption by antibiotics can lead to the development of *C. difficile* infection (CDI)^6^, the mechanisms underlying *C. difficile* expansion after microbiota disturbance are only just emerging. Both dietary and microbiota compositional changes have been demonstrated to lead to alterations in the colonic environment that favour or suppress certain enteric pathogens such as *C. difficile*^7–11^ and indeed *C. difficile* virulence has been linked to the ability to both effectively utilise nutrients in the dysbiotic gut environment^12^ and to sporulate^13^. During CDI, there is rapid expansion of the *C. difficile* vegetative cell population, with subsequent production of the two proven virulence factors, the toxins (A&B), and spores which serve as the transmissible elements^14,15^.

While a variety of model systems that are indispensable in the study of *C*. *difficile* pathogenesis have been described^16^, studies with humans are, by contrast, limited to prospective or retrospective sampling and elucidation of *C*. *difficile* strain variants. While it could be argued that many of the experimental systems that exist to study *C*. *difficile* pathogenesis have rather limited relevance to the human gut, recent work with *in vitro* continuous flow bioreactors has elegantly demonstrated the increased competitive fitness of ribotype 027 *C. difficile* strains in a mixed microbiota model^17^ and shown that microbial communities representative of key features of the gut can be cultivated and manipulated successfully^18^. Such *in vitro* models have also been used to investigate and model antibiotic exposure^19^, intestinal biofilm development^20^ and genomic stability of *C. difficile* during simulated infection experiments^21^. In other systems that seek to investigate changes within the human gut, faecal water (FW), the aqueous phase of human faeces^22^, is an attractive means of linking changes in colonic contents with gut health outcomes^23–27^. FW has been used as a biologically–relevant challenge agent in a range of gut studies^28–30^ as it contains a variety of unbound, soluble components including bile acids, fatty acids, amino acid residues and derivatives, (poly)phenols, and short-chain fatty acids^31–33^. These metabolites are likely to modulate the function and composition of the microbiome. To allow physiologically–relevant modelling of *C. difficile* under controlled culture conditions, we have incorporated FW into growth media in order to better mimic the human gut environment. Using our novel approach we now demonstrate for the first time that expression of genes essential for pathogenesis are significantly differentially expressed in the human faecal water milieu.

## Results and Discussion

### *C. difficile* cell length increases, yet overall population growth is unaffected in FW media

To create a physiologically more relevant model of the colonic milieu to study gut pathogen biology, a pooled faecal water (FW) sample was produced as previously described^32^ from two male donors (age 40+/−2 years). We characterised the FW using LC–MS^n^ and demonstrated that it contained components identified in previous investigations^32,34–36^. Some 30 FW components, many of which are known constituents of faeces (e.g. stercobilin and urobilinogen) were identified (Table 1), while others gave MS data and putative IDs consistent with previous analyses including a number of bile acid derivatives^37^ as well as some unidentified components.

**Table 1:** Components identified in faecal water.

Peak^a^	RT^b^	*m/z* [M-H]^−^	MS^2c^	Formula^d^ *m/z* [M-H]^−^	Putative Identity	Reference/Database
**1**	5.32	181.0357	138 (43) **122** (59)	C_6_H_5_N_4_O_3_	Xanthine (1-methyl uric acid)	Jiménez-Girón *et al*.^36^
**2**	5.83	131.0345	113 (18) **87** (44)	C_5_H_7_0_4_	Hydroxy-oxopentanoic acid	ChEBI:111517
**3**	9.23	145.0500	127 (18) **101** (44) 83 (62)	C_6_H_9_O_4_	Adipic acid (hexanedioic acid)	ChEBI:30832
**4**	10.15	357.0735	339 [18] 313 [44] **277** [80] 260 [97]	ND	UK (277-sulphate)	NA
**5**	10.71	195.0514	180 [15]	C_9_H_9_ON	UK	NA
**6**	11.87 + 12.13	261.0059	181 [80]	C_9_H_9_O_7_S	Homovanillic acid sulphate	ChEBI:88405
**7**	12.93	181.0497	163 [18] **137** [44] 113 [68]	C_9_H_9_O_7_	Homovanillic acid	ChEBI:545959
**8**	14.83	305.0681	**225** [80] 97 [128]	C_12_H_17_O_7_S	UK (225-sulphate)	NA
**9**	17.85	165.0549	147 [18] **121** [44] 97 [68]	C_9_H_9_O_3_	Hydroxyphenylpropionic acid	ChEBI:32980
**10**	22.27	187.0966	169 [18] **125** [62] 97 [90]	C_9_H_15_O_4_	Azelaic acid (nonanedioic acid)	ChEBI:48131
**11**	22.69	329.1007	311 [18] **285** [44] 249 [80] 205 [128] 123 [206]	ND	UK (249-sulphate)	NA
**12**	23.84	199.0964	**155** [44] 181 [18]	C_10_H_15_O_4_	Decenedioic acid	ChEBI:89730
**13**	24.11	359.1109	**315** [44] 341 [18]	C_19_H_19_O_7_	Unknown	NA
**14**	24.35	389.1215	**345** [44] 313 [68] 271 [118]	C_20_H_21_O_8_	Dehydro-deoxycholic acid	NA
**15**	24.69	413.1609	395 [18] 383 [30] **369** [44] 353 [60] 333 [80]	C_20_H_29_O_7_S	UK (333-sulphate)	NA
**16**	25.16	413.1609	395 [18] 383 [30] **369** [44] 353 [60] 333 [80]	C_20_H_29_O_7_S	UK (333-sulphate)	NA
**17**	25.40 + 26.16	201.1121	**139** [62] 157 [44]	C_10_H_17_O_4_	Decanedioic acid	ChEBI:41865
**18**	25.69	453.2824	435 [18] **407** [46]	C_25_H_41_O_7_	Cholic acid 1 (+formate)	LMST04010001
**19**	25.87	319.1642	301 [18] **291** [28] 273 [46]	C_17_H_23_N_2_O_4_	UK	
**20**	26.75	591.3143	547 [44]	C_33_H_43_N_4_O_6_	Urobilinogen	ChEBI:29026
**21**	26.92	593.3297	549 [44]	C_33_H_45_N_4_O_6_	Stercobilin	ChEBI:26756
**22**	27.84	453.2824	435 [18] 409 [44] **407** [46]	C_25_H_41_O_7_	Cholic acid 2 (+formate)	LMST04010001
**23**	29.16	485.2178	**331** [154] 259 [226]	C_27_H_33_O_8_	Bile acid derivative	NA
**24**	29.46	453.2826	435 [18] **407** [46] 391 [62]	C_25_H_41_O_7_	Cholic acid 3 (+formate)	LMST04010001
**25**	30.14 + 30.49	471.2390	391 [80] **453** [18] 427 [44]	C_24_H_39_O_7_S	Sulfodeoxycholic acid	ChEBI:88888
**26**	31.10	453.2826	435 [18] **407** [46] 373 [80]	C_25_H_41_O_7_	Cholic acid 4 (+formate)	LMST04010001
**27**	31.27	451.2667	433 [18] **407** [44]	C_25_H_39_O_7_	dehydrocholic acid (+formate)	PB 6674
**28**	32.11	471.2390	**453** [18] 421 [50] 378 [93] 373 [98]	C_24_H_39_O_7_S	Sulfodeoxycholic acid	ChEBI:88888
**29**	32.41	531.2198	513 [18], 489 [42] 471 [60] 427 [104]	C_32_H_35_O_5_S	Cyprinol sulphate-like bile acid derivative	HMDB 0006888
**30**	33.24	453.2822	435 [18] 409 [44] **407** [46]	C_25_H_41_O_7_	Cholic acid 5 (+formate)	LMST04010001

Most of these peaks were present in media at the end of the incubation. Most were unchanged during growth of *Clostridium difficile* strain 630 but some were marginally increased.

Databases: PB = PubChem (https://pubchem.ncbi.nlm.nih.gov/); ChEBI (http://www.ebi.ac.uk/chebi/); LM - LIPID MAPS (http://www.lipidmaps.org/data/structure/); HMDB (http://www.hmdb.ca/).

^a^peak designation; ^b^retention time; ^c^MS^2^ fragments in bold are the most intense; figures in brackets are neutral loss. All MS^2^ fragments apart from bold or underlined are minor fragments; ^d^predicted formulae based on *m/z* [M-H] values, UK = unknown.

**Figure 1:**
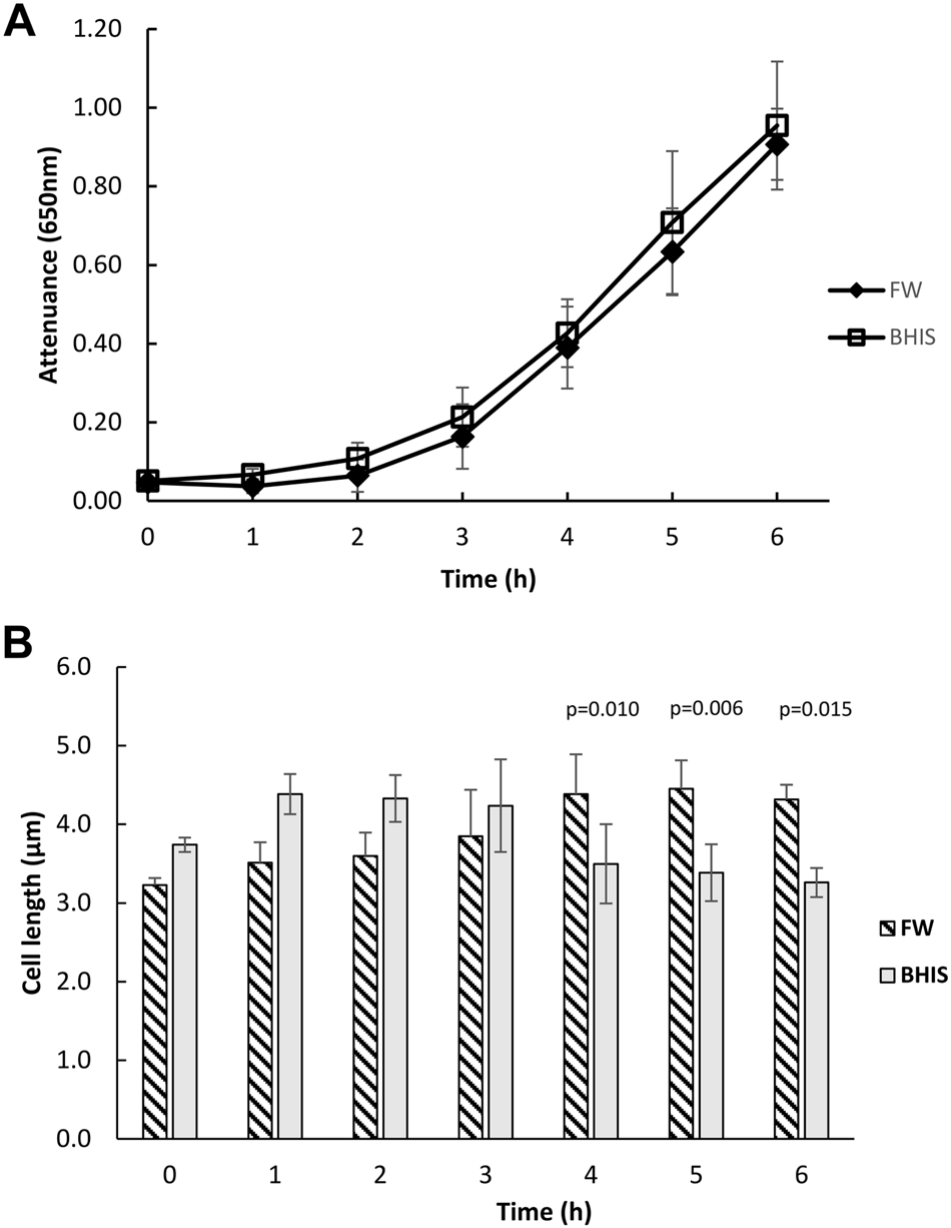
Growth of *Clostridium difficile* strain 630 in faecal water media. (**A**) Growth (D_650mn_) of *Clostridium difficile* strain 630 in faecal water media and in BHIS. Data presented are means of three independent biological replicates and error bars represent the standard deviation of the mean. (**B**) Cell lengths of *Clostridium difficile* strain 630 grown in faecal water media and in BHIS. Samples from growth curves shown in (**A**) were Gram’s stained by standard methods and 100 cells were measured per sample. The data presented are the means of 3 biologically independent experiments and error bars represent standard deviation of the mean. P values represent statistical comparison (Anova, Post hoc: Dunnett t (2-sided)) between other time points and T = 0; T4, p = 0.010, T5, p = 0.006, T6 p = 0.015.

To test the hypothesis that the presence of faecal water would change *C. difficile* physiology in a way more reflective of the *in vivo* environment, we compared growth in faecal water/BHIS growth media (“FW media”) with the BHIS control. FW media was not detrimental to growth of *C. difficile* strain 630 over 6 h (Fig. 1A), although we noted that bacterial cell length increased by almost 70% at 6 h in FW media (4.3 µm versus 3.3 µm, p = 0.015) (Fig. 1B). (Supplementary Data File 1, Tables S1 and S2)

### *C. difficile* generates novel, amino-acid derived metabolites during growth in FW media

**Figure 2:**
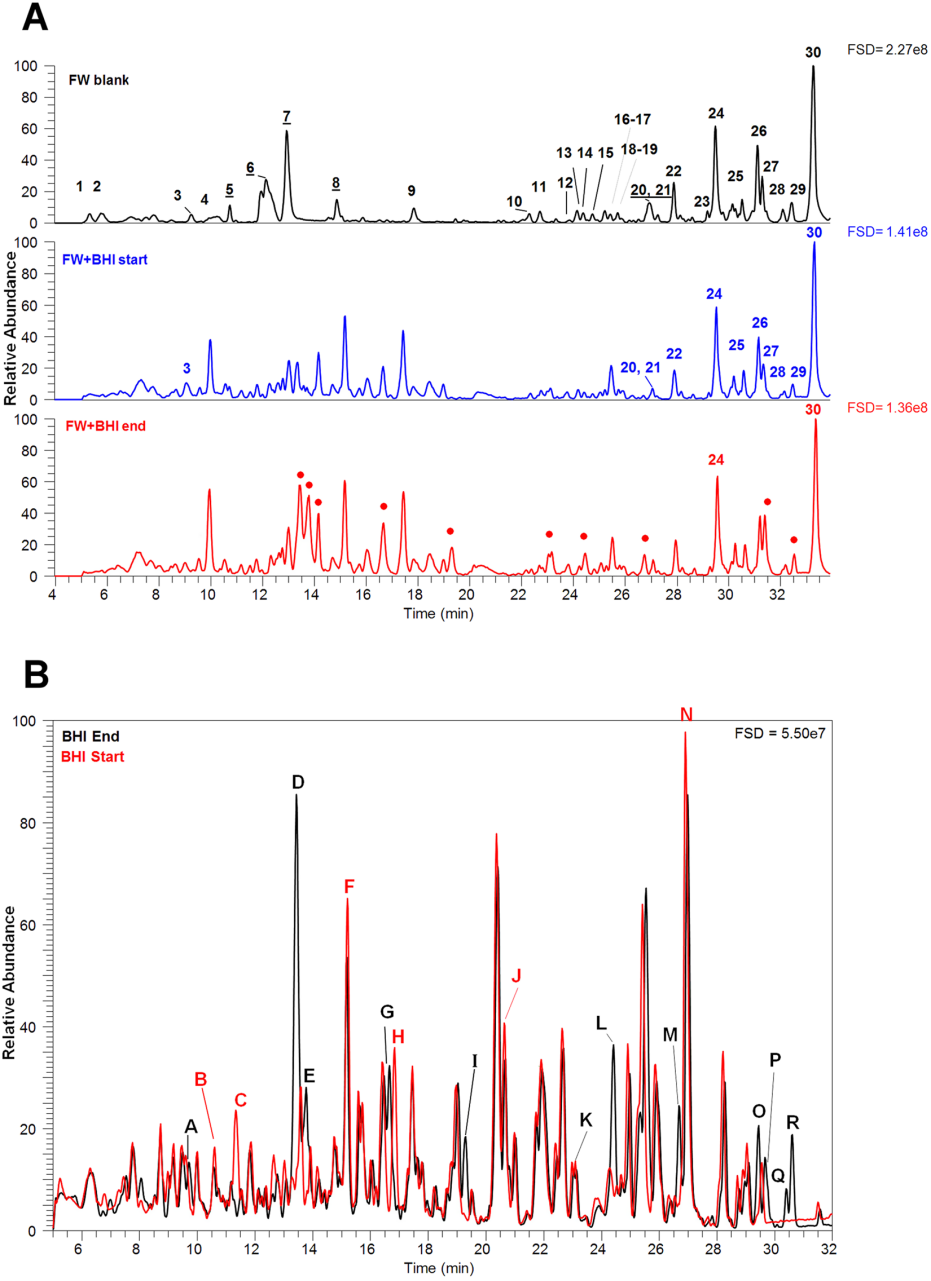
Metabolite peaks that changed during growth of *Clostridium difficile* strain 630 in faecal water media. (**A**) Metabolite peaks derived from faecal water (Identities in Supplementary Data File 1, Table S1). (**B**) Metabolite Peaks that changed during growth of *C. difficile* strain 630. Peaks A–R were noted to alter between the MS traces of the 0 h and 6 h time points while peaks 1–17 were noted as being increased during growth using the XCMS data processing method (Identities in Supplementary Data File 1, Table 2).

Having demonstrated that *C. difficile* population growth was not affected by the presence of FW components, we determined whether any of the FW components were utilised by *C. difficile* during growth, or if any bacterially–derived metabolites could be identified in culture supernatants. FW–derived components (n = 30) were not subject to further metabolism or degradation by *C. difficile* and were largely unchanged between the start and end of the 6 h incubation (Fig. 2A). The main trend was towards a slight increase in abundance during incubation (Fig. 2B, Table 2), possibly caused by release of FW components bound to BHI media constituents during growth of *C. difficile*.

**Table 2:** Identified metabolites that changed during growth of *Clostridium difficile* strain 630 in FW media.

Peak^a^	RT^b^	Comparison of change in metabolite over time (0–6 hr) between FW & BHI^c^	*m/z* [M-H]^−^	MS^2d^	Formula^e^ [M-H]^−^	Putative Identity	Database
**1**	5.18	**↑ FW** > **↑ BHI**	**144.0658**	126 (18), 116 (28), **100** (**44**), 98 (46),74 (70)	C_6_H_10_NO_3_	*N*-butyryl glycine	PC 88412
**2**	8.03	**↑ FW** > **↑ BHI**	**149.0270**	119 (30), **101** (**48**), 89 (60), 83 (66)	C_5_H_9_SO_3_	hydroxyl (methylthio) butanoic acid	PC 11427
**3**	8.88 9.97	**↑ FW** > **↑ BHI ↑FW** = **↑ BHI**	**158.0813 158.0813**	114 (44), 112 (46), 102 (56), **88** (**70**), 74 (84) 131 (27), **116** (**42**), 114 (44), 102 (56), 88 (70) 86 (72), 74 (84)	C_7_H_12_NO_3_ C_7_H_12_NO_3_	pentanoyl glycine isomers	NA
**A**	9.92	**↑FW** = **↑ BHI**	**227.1024**	183 (44)	C_10_H_15_N_2_O_4_	pyroglutamyl valine**	PC 152416
**B**	10.60	**↓ FW** = **↓ BHI**	**259.0741**	**215** (44), 211 (48), **167** (**92**)	C_18_H_11_O_2_	pyreneacetic acid	PC 186770
**4**	10.66	**↑FW** = **↑ BHI**	**181.0494**	**163** (**18**), 135 (46),119 (62)	C_9_H_9_O_4_	homovanillic acid	CS 1675
**5**	11.28	**↑FW < ↑ BHI**	**243.1696**	225 (18), 199 (44), 182(61) 163 (80), **145** (**98**), 130 (113) 128 (115)	C_12_H_23_N_2_O_3_	hexanoyl lysine	CS 7992036
**C**	11.35	**↓ FW** = **↓ BHI**	**269.0761**	**225** (**44**), 122 (147)	C_11_H_13_N_2_O_6_	UK	
**6**	11.51	**↑FW** = **↑ BHI**	**222.0756**	**180** (**42**), 178 (44), 163 (59)	C_11_H_12_NO_4_	*N*-acetyl tyrosine	PC 68310
**D**	13.45	**↑ FW** > **↑ BHI**	**131.0707**	85 (46)	C_6_H_11_O_3_	hydroxycaproic acid	PC 99824
**E**	13.79	**↑ FW** > **↑ BHI**	**131.0706**	85 (46)	C_6_H_11_O_3_	hydroxycaproic acid isomer	PC 99824
**7**	14.85	**↑ FW** > **↑ BHI**	**172.0968**	**130** (**42**), 128 (44), 88 (**84**), 74 (98)	C_8_H_14_NO_3_	pentanoyl alanine	CS 15621024
**F**	15.20	**↓ FW** = **↓ BHI**	**241.1182**	197 (44)	C_11_H_17_N_2_O_4_	UK	
**8**	16.18	**↑ FW** > **↑ BHI**	**172.0966**	154 (18), 130 (42), 128 (44), 88 (84), **74** (**98**)	C_8_H_14_NO_3_	hexanoyl glycine (*N*-caproylglycine)	CS 89859
**G**	16.66	**↑ FW** > **↑ BHI**	**165.0546**	147 (18), **119** (**46**)	C_9_H_9_O_3_	3-(4-hydroxyphenyl) propionic acid	PC 10394
**9**	16.75	**↑ FW** > **↑ BHI**	**250.1065**	206 (44), **180** (**70**), 163 (87)	C_13_H_17_NO_4_	ethyl-*N*-acetyl tyrosine	CS 12729
**H**	16.82	**↓ FW** = **↓ BHI**	**273.0862**	229 (44)	C_14_H_13_N_2_O_4_	hexahydrophenazine-1,6-dicarboxylic acid	ChEBI 132261
**10**	19.30	**↑ FW** > **↑ BHI**	**186.1126**	142 (44),130 (56), **88** (**98**), 86 (100)	C_9_H_16_NO_3_	hexanoyl alanine	NA
**11**	19.62	**↑ FW** > **↑ BHI**	**264.1221**	220 (44), **180** (**84**),163 (101) 158 (106),107 (157)	C_14_H_18_NO_4_	pentanoyl tyrosine	CS 5142226
**12**	20.12	**↑ FW** > **↑ BHI**	**145.0861**	127 (18), **99** (**46**), 97 (48) 83 (62)	C_7_H_13_O_3_	hydroxy heptanoic acid	PC 275049
**J**	20.65	**↓ FW** = **↓ BHI**	**598.2504**	390 (208), **372** (226)	C_29_H_36_O_9_N_5_	UK	
**13**	22.11	**↑ FW** > **↑ BHI**	**200.1277**	182 (18), 172 (28), 156 (44), **130** (**70**), 114 (86), 86 (114)	C_10_H_18_NO_3_	capryloyl glycine (2-octanamidoacetic acid)	CS 76040
**K**	23.15	**↑FW** = **↑ BHI**	**385.1411**	259 (126)	C_15_H_23_N_5_SO_5_	UK	NA
**L**	24.40	**↑FW** = **↑ BHI**	**278.1374**	234 (44), 260 (18), **180** (**98**) 163 (115)	C_15_H_20_NO_4_	hexanoyl tyrosine	CS 32674367
**14**	25.63	**↑ FW** > **↑ BHI**	**273.1221**	225 (18), 243 (30), **229** (**44**)	None	UK	NA
**15**	26.67	**↑FW** = **↑ BHI**	**246.1151**	228 (18), 202 (44), **198** (**48**), 154 (92), **148** (**98**)	C_11_H_20_NO_3_S	hexanoyl methionine	CS 80768
**M**	26.69	**↑FW** = **↑ BHI**	**214.1434**	196 (18), 170 (44),**116** (**98**)	C_11_H_20_NO_3_	hexanoyl valine	CS 24223294
**N**	26.91	**↓ FW** = **↓ BHI**	**740.4290**	**683** (**57**), 587 (152), 439 (301), 414 (326)	C_46_H_60_O_8_	UK	NA
**16**	27.47	**↑FW** = **↑ BHI**	**248.1273**	204 (44),**164** (**84**),147 (101) 112 (136)	C_14_H_18_NO_3_	pentanoyl phenylalanine	CS 14753835
**17**	27.71	**↑FW** = **↑ BHI**	**287.1377**	243 (44),**203** (**84**),158 (129)	C_16_H_19_N_2_O_3_	pentanoyl tryptophan	CS 16818921
**O** **P**	29.43 29.76	**↑FW** = **↑ BHI**	**228.1588**	210 (18),184 (44), **130** (**98**)	C_12_H_22_NO_3_	hexanoyl leucine/isoleucine	CS 20041531
**Q**	30.40	**↑FW** = **↑ BHI**	**301.1533**	283 (18),257 (44) **203** (**98**) 172 (129)	C_17_H_21_N_2_O_3_	hexanoyl tryptophan	CS 53673714
**R**	30.62	**↑FW** = **↑ BHI**	**262.1430**	218 (44), **164** (**98**) 147 (115)	C_15_H_20_NO_3_	hexanoyl phenylalanine	CS 3440214

**Figure 3:**
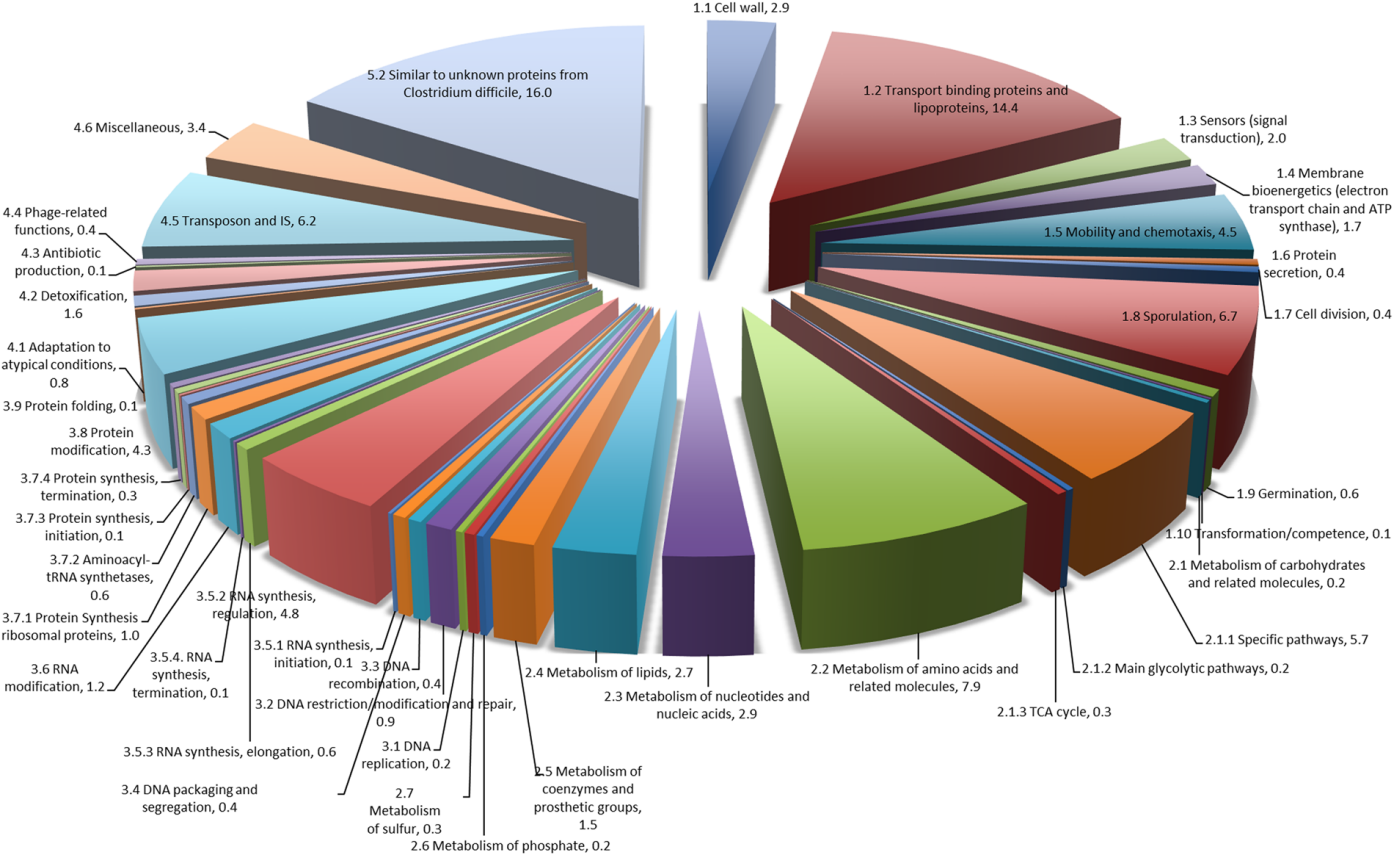
Functional categorisation of differentially–expressed transcripts in the *Clostridium difficile* strain 630 faecal water transcriptome.

Databases - PB = PubChem (https://pubchem.ncbi.nlm.nih.gov/); CS = Chem Spider (http://www.chemspider.com/)., ChEBI (http://www.ebi.ac.uk/chebi/) Peaks A – R are featured in Fig. 3.

**Identified in faecal water^95^

^a^peak designation; ^b^retention time; ^c^arrows denote whether the compound increases or decreases during the growth period; <, > and + denote if the increase or decrease was more apparent in the + FW or BHI alone incubations; ^d^MS^2^ fragments in bold are the more intense, figures in brackets are neutral loss. All MS^2^ fragments apart from bold or underlined are minor fragments; ^e^predicted formula^e^ based on *m/z* [M-H] values, UK = unknown.

We focused on metabolites whose abundance consistently increased during incubation, using the XCMS process with data checking to eliminate low abundance peaks, adducts and multiply charged ions (see Supplementary Data File 1, Figs S1–S6). Overall, there was an increase in certain components, most notably a set of amino acids esterified to hexanoic and pentanoic acids including glycine, lysine, alanine, tyrosine, methionine, valine, phenylalanine, tryptophan and leucine and/or isoleucine. No threonine, arginine, histidine, asparagine, aspartate, cysteine, glutamine, proline or serine hexanoyl derivatives were found. These putative hexanoyl amino-acid derivatives yielded characteristic MS^2^ fragments which suggested fragmentation at the amide bond to produce the M^[−H]^ ion of the amino acid and a neutral loss of 98 atomic mass units (amu), which could be due to the aldehyde, hexenal (C_6_H_10_O). In a similar manner, fragmentation of pentanoyl derivatives produced the M^[−H]^ ion of the amino acid and a neutral loss of 84 amu due to the aldehyde, pentenal, (C_5_H_8_O; 14 amu less than hexenal). Other common fragmentations can be assigned (i.e. neutral loss of 42 (C_3_H_6_ = propene), neutral loss of 56 (C_4_H_8_ = butene), neutral loss of 70 (C_5_H_10_ = pentene) and are consistent with such structures. Other neutral losses such as 44 and 46 amu, respectively, can be ascribed to CO_2_ and formic acid from the amino acid components. These putative hexanoyl and pentanoyl amino acid derivatives have not previously been identified in cultures of *C. difficile* but this microorganism is known to produce isocaproic acid (also known as isohexanoic acid) during growth and the accumulation of this C6 fatty acid has previously been used as a diagnostic for *C. difficile* in stool samples^38,39^. Investigations into biofuel production by Clostridia have shown that hexanoyl–coA is a key metabolite for the production of hexanol^40^ and the formation of these putative hexanoyl and pentanoyl amino-acid derivatives may be a consequence of growth of *C. difficile* in the amino acid–rich BHI media. Earlier work showing isocaproic acid accumulation^38,39^ used less sensitive GC–MS techniques that required sample derivatisation and thus would not have detected the amino acid derivatives we identified. Of all the metabolites whose abundance increased during growth of *C. difficile* (Table 2), only hexanoyl lysine (peak 5, −*m/z* 243) was higher in the BHI media than in FW media (Supplementary Data File 1, Fig. S5). In fact, growth of *C. difficile* in the presence of FW did not reduce the levels of any of the other putatively–identified novel components (i.e. six components were substantially increased and four components marginally increased).

### Considerable modulation of the *C. difficile* transcriptome results from growth in FW media

**Figure 4:**
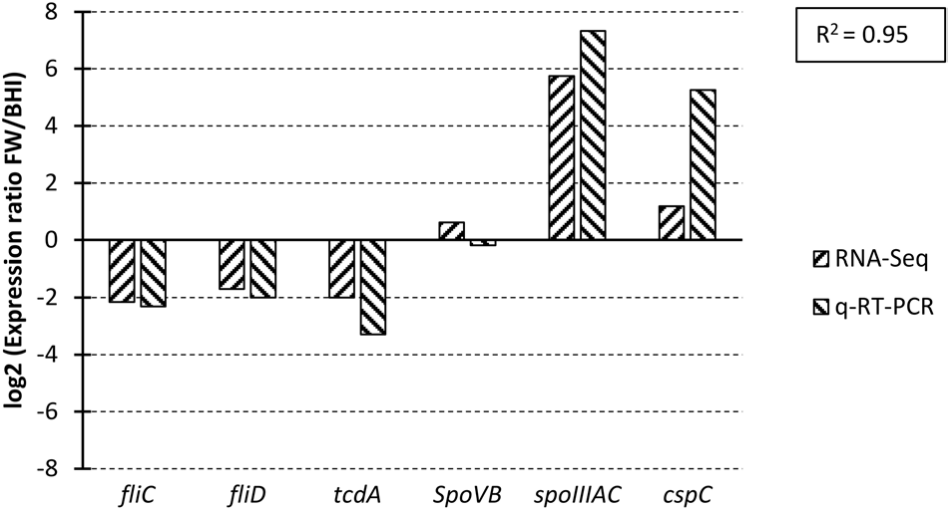
Comparison of qRT-PCR and RNAseq data for selected *Clostridium difficile* strain 630 genes. For each individual gene, expressional changes determined by RNAseq (up-hatched columns) and by qRT-PCR (down-hatched columns) are shown relative to the BHIS control, and show good correlation between the two datasets (R^2^ = 0.97). *rpsJ*, *gyrA* and *adk* were used as reference genes.

Some 889 *C. difficile* strain 630 transcripts were differentially expressed (DE) (padj < 0.001, fold–change (FC) > 1.45) as a result of exposure to faecal water with 497 (56%) exhibiting increased expression and 392 (41%) being decreased (Supplementary Table S3). The largest numbers of DE genes were in categories “Similar to unknown proteins” (16%), “Transport binding proteins and lipoproteins” (14.4%), “Metabolism of amino acids and related molecules” (7.9%), “Transposon and IS function” (6.2%), “Sporulation” (6.8%), “Specific metabolic pathways” (5.7%), and regulation of RNA synthesis (4.8%) (Fig. 3). Orthogonal validation of expressional changes using qRT-PCR showed good correlation (R2 = 0.95) between RNAseq and qRT-PCR data (Fig. 4) (Supplementary Data File 1, Table S4) when applied to a number of motility and sporulation genes.

**Figure 5:**
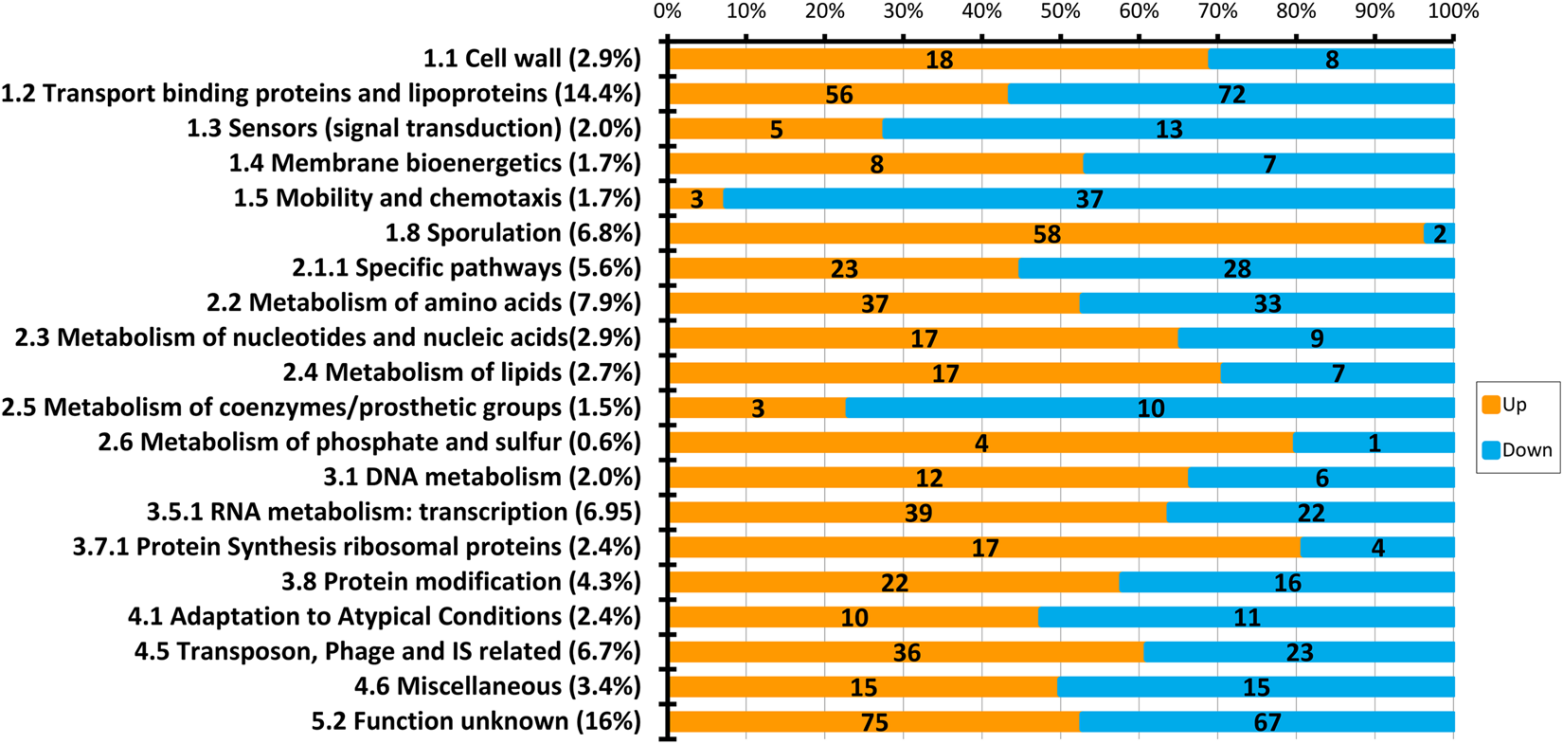
Differentially–expressed genes in the *Clostridium difficile* strain 630 faecal water transcriptome by functional category. Orange – increased expression; blue – decreased expression, p < 0.001. Analysis was conducted only on functional categories within which > 5 genes were differentially expressed.

**Figure 6:**
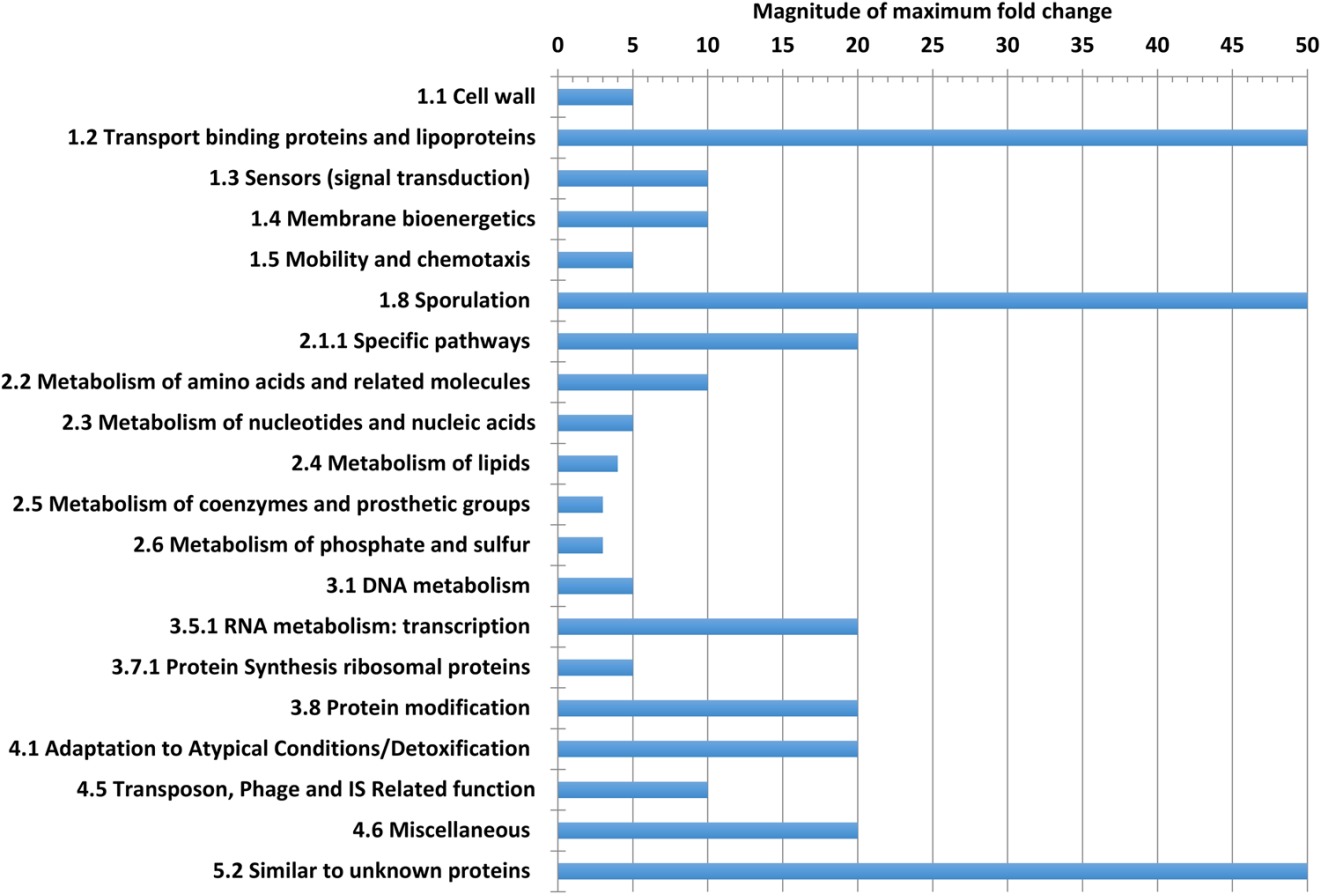
Maximum fold–changes in gene expression within selected functional categories. Functional Categories with less than five differentially expressed genes were not included in the analysis. Transport, sporulation and conserved hypothetical protein–encoding genes exhibited the largest expressional changes in response to faecal water media.

We noted that within the 889 DE genes, a slightly larger proportion exhibited increased expression in FW media with the exception of those in the categories of signal transduction, motility, genes associated with specific pathways, metabolism of co–factors, and detoxification (Fig. 5). Considerable expressional changes in the transcriptional programme of *C. difficile* strain 630 were apparent in regard to genes associated with sporulation, protein synthesis and protein modification. Nine sporulation–associated genes exhibited > 100–fold increases in expression however the largest absolute increase in expression (445–fold) was in CD1065, which encodes a 146 amino acid ‘conserved hypothetical protein’. A number of investigations into *C.difficile* sporulation have indicated that CD1065, and indeed a number of genes encoded by CD1063A-CD1067, are strongly regulated by either σ^E^ or σ^K^, with published data indicating that CD1065 is strongly induced by σ^E^ in the mother cell during sporulation^41–43^. The largest decreases in expression (>100–fold) were in a three–gene ATP binding cassette (ABC) transporter operon encoded by CD0873–0875. The largest fold–changes in gene expression were found in the categories of (i) transport, binding proteins and lipoproteins (up to 270–fold), (ii) sporulation (up to 300–fold), and (iii) genes encoding hypothetical proteins (up to 445–fold). Genes involved in specific metabolic pathways, RNA metabolism, protein modification, adaptation to atypical conditions and those categorised as miscellaneous exhibited fold changes of no more than 20–fold. The least extensively–changed genes were those involved with metabolism of phosphorus, sulphur, lipids, and coenzymes (Fig. 6), indicating that these central metabolic pathways were relatively unperturbed in the presence of FW. We have previously demonstrated apparent robustness of *C. difficile* central metabolic pathways under mild heat stress^44–46^, however the extreme perturbations in sporulation, transport and conserved hypothetical protein–encoding genes led us to consider these biological processes, and their implications for the lifestyle of this important pathogen, in detail.

### *C. difficile* sporulation gene expression increases during growth in FW media

In the model Gram positive organism *Bacillus subtilis*, and also in *C. difficile*, sporulation is initiated by a two–component system with Spo0A and associated kinases^47,48^, leading to the sequential, compartment–specific activation of the strictly conserved sporulation–specific sigma factors, σH (early), σF, σE, σG, and σK (late)^47,49^. Thereafter, however, differences have been shown in the order, activation, and function of the sigma factors in *C. difficile*^50^. Genome–wide mutational analyses of sigma factor function in *C. difficile* have revealed that while their transcriptional and functional sequence (*sigE* & *sigF* – early, *sigG* & *sigK* – late) is broadly conserved with the *B. subtilis* model, there are differences in the *C. difficile* developmental programme^51^. Spores were not visible in cultures of sigma factor mutants, indicating their critical role in the various stages of sporulation and in the production of mature, heat-resistant spores^42^. Intriguingly, and in contrast to *B. subtilis*, the activity of σE was partially independent of σF; σG or σK did not require σE or σG, respectively and *sigG* transcription was not dependent on σF^41,42^. Taken together, the published data suggests minimal intercompartmental communication and a weaker connection between forespore and mother cell^50^, in addition to a looser association between gene expression and morphology in *C. difficile*^41,51^. While sporulation in *C. difficile* is more akin to *B. subtilis* than to other Clostridia, it at the same time represents a more ancestral, less tightly–controlled sporulation programme that facilitates a degree of population heterogeneity during infection^52^. The recent work of Browne *et al*.^53^ showed extensive sporulation ability within the human gut microbiota, with many taxa present in the spore form. Animal models of *C. difficile* infection have revealed that in mouse, *C. difficile* spores germinate by 6 h post–infection, leading to pathogenic lesions and heat–resistant spores – comprising up to 20% of the total cfu – being detectable 24–36 h post–challenge. Thus, while sporulation under infection conditions takes some time) with *C. difficile* strain 630^15^, the process happens as early as 6 h post–infection with the ribotype 027 strain, *C. difficile* R20291^54^. Temporal examination of *C. difficile* 630 gene expression during infection in mouse^55^ and pig ligated loop models^56^ has shown that many genes associated with host adaptation, all stages of sporulation and a diversity of genes encoding “hypothetical proteins” were expressed at increased levels *in vivo*, indicating their importance in the infection process and the requirement for extensive remodelling of the transcriptome during infection. We thus hypothesised that FW would induce sporulation in *C. difficile* and sporulation genes were indeed some of the most differentially expressed in FW medium, with increases up to 300–fold. Of 60 DE genes in our dataset that had a predicted or known role in sporulation, only two exhibited decreased expression (CD2273, a ‘putative sporulation integral membrane protein’–possibly under σE control, and CD3669, a ‘putative exported protein’–part of the mature spore proteome). Of the remaining 58 genes, the expression of 22 of these was increased > 50–fold, indicating that FW is a potent inducer of sporulation genes in *C. difficile* strain 630. *C. difficile* sporulation has been extensively mapped, allowing definition of genes under control of Spo0A (CD1214, increased by 2.5–fold)–the master regulator of sporulation–and the identification of potential links between sporulation and other phenotypes^41,42,52,57^. Phosphorylation of the Spo0A protein initiates a sigma factor cascade that, acting in both mother cell and forespore, influences expression of the sporulation–specific sigma factors σF (CD0772, 9–fold up), σE (CD2643, 23–fold up), σG (CD2642, 40–fold up) and σK that control expression of early (*Bacillus* stage II and III) and late (*Bacillus* stage V and VI) sporulation genes^50^. During sporulation, a septum results in asymmetric division of the bacterial cell and creates two unequally–sized compartments. The smaller–the forespore–develops into the spore, while the larger compartment prepares the forespore for dormancy^42,51^. Taken in the context of previous identification/analysis of sporulation–associated genes^48,53^ our data indicates that, at the point of harvest, FW–grown *C. difficile* 630 cells are physiologically at what in *B. subtilis* would be categorised as stage III of sporulation, i.e. the point at which engulfment of the forespore has occurred, but prior to cortex formation. Thus, genes including the *spoIIIAA–spoIIAH* operon (all >50–fold increased), in addition to *spoIIIJ* (oxaA1, 1.6–fold increased), *spoIIID* (56–fold increased) and *sigG* (40–fold increased) exhibited considerably increased expression in FW–grown cells. Of the sporulation–associated sigma factors, *σE* exhibited the second–largest expressional increase (23–fold) in FW. The σE protein acts on a number of genes in the Clostridial sporulation cascade^41,42^ and we noted increased expression of σE–controlled genes including *spoIIID*, *spoIVA* (57–fold up), *cspBA* (22–fold up) and *cspC* (2.2–fold up). Furthermore, we observed increased expression of genes encoding certain spore coat proteins, including *cotE* (CD1433, 29–fold up). The peroxiredoxin and chitinase activities of CotE contribute to pathogenesis by facilitating degradation of gut mucus during infection^58,59^.

It has been demonstrated that decreased *oppABC* (CD0853–855, encoding an oligopeptide transporter) expression leads to earlier expression of sporulation–associated genes^13^ and the observation that *oppABC* expression was 50% lower in FW appears consistent with our other observations of FW–induced changes to the *C. difficile* transcriptome. Taken together, therefore, our gene expression data indicates that *C. difficile* cells are induced by FW components towards sporulation more rapidly than cells grown in BHIS media. The spores are the transmissible, resilient, and infectious form of the organism^14^ and thus our observation has clear implications for pathogenesis and transmission of the disease, in addition to being entirely consistent with observations by other researchers of extensive sporulation within the gut microbiota *in vivo*^15,53–56^.

### A variety of *C. difficile* transport systems are differentially expressed in FW media

In previous work we showed that phosphotransferase (PTS) sugar transport systems were largely unperturbed by heat stress^45^. By contrast, our current investigation revealed considerable changes in transporter gene expression. The PTS is the major bacterial carbohydrate assimilation system for hexoses, hexitols and disaccharides and consists of two general components–enzyme I (EI), and the histidine phosphocarrier protein (HPr)–in addition to sugar specific permeases (enzymes II) in the cell membrane. In FW media, expression of the gene encoding the EI component (CD2755) common to, and essential for, all phosphotransferase systems in the cell, was increased by 1.58–fold. In contrast, expression of the HPr kinase/phosphorylase (CD3409) that phosphorylates the cytoplasmic phosphocarrier protein Hpr at Ser42, and which also leads to activation of the LacI family carbon catabolite repressor, *ccpA*^60^, was 1.8–fold lower. Consistent with these observations, the gene encoding the IIABC component of the PTS system for uptake of beta-glucosides (*bglF*, CD0388) was increased, as was the downstream gene *bglA* (CD0389) encoding 6-phospho-beta-glucosidase, reflective of the likely increased availability of such glucoside substrates^61^ in the FW media. In addition, expression of the sorbitol specific IIB component, *srlEa*, (CD0765) was increased as was expression of CD2269 encoding the fructose specific IIABC component, *fruABC*, as were genes encoding the IIA and IIB components of the glucose PTS transport system (CD2512, CD2510, respectively). Expression of the IIC and IID components of the mannose/fructose/sorbose transport system (CD3277, CD3276) were increased by 4– and 6.6–fold, respectively. Conversely, expression of PTS system components associated with uptake of xylosides (*xyl* and *xyn* operons, CD3064–CD3070) was lower in FW, while expression of the associated transcriptional regulator (xylose repressor, *xylR*, CD3066), which functions to reduce expression of genes for uptake and metabolism of xylose, was increased. These diverse perturbations in expression of carbohydrate transport–associated genes most likely underpin an adaptive response of *C. difficile* to additional carbon sources and other diet derived metabolites present in the FW. However, the PTS is also a signalling device which has been linked to chemotaxis and regulatory functions associated with C, N and P metabolism and to the virulence of *C. difficile*^62,63^. The complex interplay between a variety of cellular systems (sugar transport, carbon catabolite repression, quorum sensing and amino acid metabolism), controls toxin production. It is known, for example, that butyrate stimulates toxin production^60^ but, in FW–grown cells we noted lower expression of 12 genes associated with carbohydrate fermentation to butyrate. The likely reduction in metabolic flux towards butyrate is consistent with the 4–fold lower expression of *tcdA* (CD0663) observed in FW (Fig. 4). Other genes encoded by the pathogenicity locus are not discussed here as our *padj* cutoff value precluded their inclusion in the list of statistically significant DE genes.

A number of ABC transporter–encoding genes were DE in FW, including some associated with transport of sugar phosphates, vitamins, oligopeptides, amino acids and also transporters associated with multidrug efflux mechanisms^64^. The most downregulated gene (270–fold lower in FW) in our dataset was an ABC transporter ‘substrate-binding lipoprotein’ (CD0873), recently identified as an adhesin that enables *C. difficile* to bind Caco–2 cells^65^. Our data suggests that, in FW at the point of cell harvest and possibly at later stages in the infection cycle in a host, *C. difficile* exhibits reduced binding to epithelial cells, consistent with increased sporulation and lowered motility. This physiological state could thus facilitate evacuation of the bacterial population from the host. Ten different lantibiotic/multidrug ABC transporters were also DE. Four exhibited increased expression–CD0161 (4.73–fold); CD1349/50 (5.3, 2.9–fold); CD2210/11 (3.7, 2.5–fold); CD2406/7/8 (all 2.58–fold up). The precise function of such transporters has not yet been defined, and consequently genomic annotations are a “general function prediction” only. Nonetheless, within the intestine, *C. difficile* and other gut pathogens must contend with innate host defences including cationic antimicrobial peptides (CAMPs, e.g., nisin) produced by both host and indigenous microbiota^64,66^. McBride and Sonenshine^67^ have shown that proteins encoded by CD1349/CD1350 are involved in resistance to CAMPs, proposing the designation *cprABC-cprK* for CD1349 to CD1352. In FW–grown *C. difficile* cells, expression of CD1349 and CD1350 (*cprA, cprB*, encoding the ATP–binding protein and the permease respectively) were increased by 5.3– and 2.9–fold, consistent with our hypothesis that increased levels of host or microbiota–derived antimicrobial peptides present in FW lead to increased expression of this specific mechanism.

### Expression of motility genes is decreased in FW media

Bacterial flagella are self–assembling molecular machines^68,69^, with flagella and type-IV pili comprising motility devices essential for the pathogenesis of certain bacteria^70,71^ including a variety of motile enteropathogens^72^. Flagellar biosynthesis is a highly–ordered process in which hierarchal control of gene expression ensures that synthesis of late–stage components is repressed until assembly of earlier components is complete^73^. Thus, only when the basal body and motor machinery is in place, do late–stage genes, including flagellin (*fliC*) become expressed. Expression of motility genes in *C. difficile* is regulated by a sigma–28 factor encoded by CD0266 (*fliA*, or *sigD*) whose expression was 1.5–fold decreased in FW–grown cells. El Meouche *et al*.^74^ (2013) demonstrated that sigD acts as a positive regulator of both flagella and toxin gene expression in *C. difficile*. Decreased expression of *sigD* could therefore be partially responsible for the reduced expression of motility genes. In FW–grown *C. difficile*, expression of genes located broadly in the F3 loci (CD0245–CD0271), including those encoding components of the basal body, motor, hook and rod, exhibited 1.5– to 2–fold decreased expression. Gene expression in the F1 locus (CD0226–CD0240) was reduced between 2– and 5.7–fold, as would be expected if these gene products were not required until assembly of the basal body was complete. In addition, expression of *fliC* (CD0239) was decreased by 4.4–fold to ~20% of the level in the control, an observation corroborated by our qRT-PCR data (Fig. 4). *flgN* (CD0230) expression was reduced by 5.7–fold and we noted likewise that expression of genes in the interflagellar F2 locus (CD0240–CD0244) decreased by just over three–fold in FW. Levels of transcript (Supplementary Table S3, base mean values), for class I flagellar genes were higher than those for the class II genes, with genes in the interflagellar locus expressed at a yet lower level still, which is logical from an assembly perspective. Flagellar operon gene expression, and thus motility of *C. difficile*, decreases in FW concomitantly with increased expression of sporulation–associated genes. The precise role of flagella in *C. difficile* pathogenesis is still unclear however, depending in many cases upon the strain tested^55^. Decreased FliC expression under clinically–relevant heat stress^44–46^, may enable better adherence to epithelial cells, a hypothesis supported by the work of Dingle *et al*.^72^ who assessed *fliC* and *fliD* disruption mutants, concluding that flagellar motility *per se* did not contribute to adherence to epithelial cells *in vitro*. Indeed, they argued that flagella were either not necessary for virulence, or that repression of motility could be a pathogenic mechanism. In a *C. difficile dnaK* mutant, lack of motility was underpinned by a 4–fold decrease in *fliC* expression with the mutant also exhibiting significantly enhanced biofilm–forming ability^75^. Other mutational studies have also shown that non–flagellated *C. difficile* cells exhibit lower levels of toxin production^76^ in addition to increased sporulation as a result of the pleiotropic role of FliC in *C. difficile* gene regulation^77^.

In addition to changes in flagellar operon gene expression, we noted a 2– to 3–fold increase in expression of some genes in the secondary type IV pilus (TFP) locus. This increased expression of genes encoding a type–IV pilin, an associated type–II secretion system protein, and a pilus assembly ATPase (CD3294-6) suggests that pilus–driven motility may possibly be more important in a FW milieu and in certain stages of the infection process. Regardless of the role of TFP during infection, bacterial flagella are known to promote intestinal lesions via host inflammatory responses: *C. difficile* FliC protein recognizes TLR5 and consequently activates the NF-κB and the MAPK signalling pathways that elicit synthesis of pro–inflammatory cytokines^78^. Such host receptors are not present in our experiment and decreased expression of flagellar loci may represent interplay between a putative motility phenotype and an adhesion, or indeed sporulation, phenotype. Nonetheless, flagella are energetically extravagant structures and, in the challenging environment of the gut, it makes strategic sense for motility in a semi–solid milieu to be driven by less resource–intensive structures such as type–IV pili.

### Many Genes encoding Conserved Hypothetical Proteins are differentially expressed in FW media

The largest number of DE genes (n = 141) were placed in the ‘Similar to unknown proteins’ category and 19 of these had expressional changes of > 20–fold in FW–grown cells. None of the proteins encoded by these genes had predicted signal peptides^79^ and with the exception of CD1726 and CD3522, were all predicted by SecretomeP^80^ to be non–classically secreted. Nine of the gene products had PsortB^81^–predicted locations in the cytoplasmic membrane and the majority of these 141 proteins possessed no conserved domains that might indicate their potential function. Nonetheless, literature and database interrogation allowed us to link many to a role in sporulation. The most downregulated conserved hypothetical protein–encoding gene was CD2344, which has been identified as a putative succinate transporter with a role in *C. difficile* gut colonisation^12^. We have also shown here that expression of a variety of other genes in the succinate to butanoate fermentation pathway–which lie transcriptionally downstream of CD2344 in the same operon structure^82^ (e.g. *cat1*, *sucD*, *abfD*, and *cat2*, *4hbd*)–were decreased by ~4–fold in FW. A number of genes reported to be regulated by sporulation–specific sigma factors^41–43^, including σK (CD3580 & CD1065), σG (CD2808 & CD2375) and σE (CD1063A-C, CD2150A & CD3522) were also DE in FW. Dembek^83^ reported that a large proportion of *C. difficile* spore transcripts encoded proteins of unknown function and proposed that these were indicative of the difference between the transcriptional programme of vegetative cells and spores. Three such genes exhibited increased expression in FW–CD3551A (71–fold), CD2374 (30–fold) and CD2229 (36–fold). In addition, DE genes including CD1929, CD1884, CD2657, CD2374 and CD2375 were also reported by Janoir *et al*.^55^ to be expressed at higher levels in stationary phase *C. difficile* 630 cells at 14 h and 38 h–i.e. where the sporulation process would be well–established.

## Conclusions

We set out to establish a new means of investigating gut pathogen biology *in vitro*. LC-MS^n^ metabolomic analysis of FW allowed us to identify 30 individual components including urobilinogen, stercobilin and several cholic acid derivatives. Having established that the FW was–metabolomically–consistent with previous reports, we demonstrated that in the presence of FW, growth of *C. difficile* strain 630 was largely unaffected, save for an increase in cell length that our transcriptome data indicates is most likely a prelude to sporulation. A primary question was whether *C. difficile* strain 630 could utilise components of FW. Our analysis showed that while FW metabolites were not further metabolised during growth, a set of previously unknown *C. difficile*–derived hexanoyl– and pentanoyl– derivatives of amino acids were produced. These metabolites are not only novel biomarkers for the presence of this pathogen, but also reflect previously unrecognised metabolic capabilities within *C. difficile* strain 630. RNA sequencing showed clearly that the primary transcriptomic response of *C. difficile* strain 630 to FW was an acceleration of the sporulation cascade. FW–grown cells exhibited increases of up to 300–fold in the expression of sporulation–associated genes, with concomitant decreases in motility and toxin gene expression. These changes are reflective of the interplay between FW components and the expression of sensor kinase/response regulator systems, and transcription factors, many of which exhibited increased expression in FW–grown cells. Interestingly, none of the classical stress–response genes were differentially expressed, supporting the rationale that *C. difficile* adapts easily to a faecal milieu. The considerable modulation of a variety of transport systems is consistent with the addition of FW components to the growth media. Overall, therefore, our *ex vivo* FW model represents a new and unique means of assessing the response of *C. difficile* strain 630 to gut metabolites allowing us to describe, for the first time, the faecal milieu–associated physiological changes in this important pathogen.

## Materials and Methods

### Chemicals and Glassware

All chemicals and reagents of Analar grade or better were purchased from Sigma-Aldrich (Poole, UK) unless stated. Brain Heart Infusion (BHI) agar and broth and yeast extract were purchased from Oxoid (Basingstoke, UK). All molecular biology reagents were purchased from Invitrogen (Renfrewshire, UK) save for qPCR reagents, which were obtained from Roche Diagnostics (Hertfordshire, UK) and random primers, which were obtained from Promega (Southampton, UK). Lysing Matrix A tubes were from MP Biomedicals (Cambridge, UK) and all glassware was cleaned with 1% Virkon (Antec Intl. Ltd., UK) overnight prior to steeping in 2% Decon (Decon Labs Ltd., UK) for 4 h prior to use.

### Preparation of faecal water for inclusion in BHIS media

The Ulster University Research Ethics Committee exempted this study from review because donors were not involved in any intervention; the samples received were not collected by means of intervention and were used solely for preparation of a bacterial growth media. Written consent was obtained for provision of the donor faecal samples. Fresh faecal samples were provided by two apparently healthy individuals (2 males, age range 38–42 years, who had not taken antibiotics within the previous three months). Stool samples were collected from donors and stored at 4 °C for up to 2 h before processing. Faecal water was produced as described in Gill *et al*.^32^. In brief, the faecal sample was weighed, mixed 1:1 wt/vol with ice cold 0.01 M phosphate buffered saline (PBS) then homogenised in a Seward 600 stomacher (2 × 2 min cycle). The resultant faecal slurry was centrifuged at 50,000 × g for 2 h at 4 °C in the 70.1Ti rotor of a Beckman L8-M centrifuge and the supernatant removed and filter–sterilised (0.22 μm filter) on ice before being aliquoted for storage at −70 °C.

### Growth of *Clostridium difficile* strain 630

*Clostridium difficile* 630 (ATCC no. BAA-1382D-5) was grown under anaerobic conditions in a Don Whitley MACS MG500 anaerobic cabinet (Don Whitley Scientific Ltd, Yorkshire, UK) using a single gas mix (BOC, UK) of 80% N_2_, 10% CO_2_ and 10% H_2_, at 37 °C. Standard growth media was brain heart infusion broth supplemented with 5 g L^−1^ yeast extract and 1 g L^−1^ L-cysteine (BHIS). For media containing faecal water, 2–fold concentrated BHIS (50 mL) was prepared and a 50 mL aliquot of filter–sterilised FW added to this aseptically post–autoclaving, resulting in 1 × BHIS containing 50% FW (“FW media”). Control media was prepared from 2–fold concentrated BHIS to which was added an equal volume of sterile PBS. Three starter cultures of *C. difficile* 630 were set up in 20 mL of FW media in glass universal bottles and each was inoculated with a single colony of freshly grown *C. difficile* 630 from a BHIS agar plate. Starter cultures were incubated overnight and used to inoculate fresh media, in triplicate, at 5% (vol/vol). Growth was recorded hourly as attenuance at 650 nm (*D*_650nm_)^44^ against un–inoculated BHIS and FW media references. Multiple cell pellets were collected by centrifugation from all six cultures at mid–log phase of growth (*D*_650nm_ = 0.6). Culture supernatants were removed to fresh tubes and both cell pellets and supernatants were placed briefly in liquid nitrogen before immediate transfer to −70 °C until required.

### RNA extraction and quality control

RNA extraction was via a Qiagen RNeasy® Mini kit with the addition of a mechanical lysis step as described previously^45^. RNA was checked for absence of DNA contamination by PCR with *gyrA*, *rpsJ*, and *adk* primers (Table 3) followed by agarose gel electrophoresis and imaging under UV light. A Nanodrop™ 1000 spectrophotometer (Thermo Scientific) was used to quantify the amount of RNA in the samples and integrity of total RNA was then determined using an RNA 6000 Nano Assay kit with an Agilent 2100 Bioanalyzer (Agilent Technologies, CA, USA) instrument as per the manufacturer’s instructions. Only RNA samples with RIN > 9.0 were used in subsequent procedures.

**Table 3:** PCR primers.

Gene	Locus	Description	Primer	Sequence (5′ → 3′)	Binding position	Product size (bp)	Annealing temperature (°C)	Reference
*rho*	CD3487	Transcription termination factor Rho	rho-F rho-R	CATCAAGCAATAAATCATCTC CTGGTTCTAGGATGGATGATG	141–293	153	57	Metcalf *et al*.^96^
*gyrA*	CD0006	Gyrase subunit A	gyrA-F gyrA-R	CTCGTATTGTTGGGGACGTT ATCCCCATCAACAGAACCAA	197–342	146	57	Denève *et al*.^97^
*adk*	CD0091	Adenylate kinase	adk-F adk-R	GTGTATGTGATGTATGCCAAG CCTAAGGCTGCGACAATATC	443–638	196	57	Metcalf *et al*.^96^
*rpsJ*	CD0072	30 S ribosomal protein S10	RpsJ-F rpsJ-R	GATCACAAGTTTCAGGACCTG GTCTTAGGTGTTGGATTAGC	101–251	151	57	Metcalf *et al*.^96^
*groES*	CD0193	10 kDa chaperone	groES-F groES-R	AGTTTTACCAGGAGCAGCTAAAG CCTTATCTCCCACTGTCAATTCC	75–190	116	57	This study
*groEL*	CD0194	60 kDa chaperone	groEL-F groEL-R	TTGCTGGAGGAGTAGCTGTTG AAAAGCAGTTCCTCCACCAG	1109–1251	143	57	This study
*fliC*	CD0239	Flagellin subunit	fliC-F fliC-R	TGATGATGCTGCTGGACTTG ACGAACCTTCTGCTGTTTGTAC	120–238	119	57	This study
*fliD*	CD0237	Flagellar cap protein	fliD-F fliD-R	AGCTGGACAAATTGCCAGTG CCTTGGTCATCAGTTACATCAGC	621–734	114	57	This study
*tcdA*	CD0663	Toxin A	tcdA-F tcdA-R	AGCTTTCGCTTTAGGCAGTG ATGGCTGGGTTAAGGTGTTG	1121–1250	129	57	This study
*spoVB*	CD3498	Stage 5 sporulation protein B	spoVB-F spoVB-R	ATTCAGGGAATGGGAAAACC TTAATCATGGCTGCCACAAA	1165–1325	161	57	This study

### Transcriptome sequencing

RNA sequencing (RNAseq) and initial bioinformatics analysis was performed at Deepseq (University of Nottingham, UK). RNA samples were shipped to Deepseq on dry ice and upon receipt, total RNA quality was once more assessed using the Agilent RNA 6000 Nano Kit (Agilent Technologies, 5067–1511) on the Agilent 2100 Bioanalyzer. The total RNA concentration was measured using the Qubit RNA BR assay kit (Life technologies, Q10210). A 1 µg amount of Total RNA was used for rRNA depletion using the Ribo-Zero rRNA Removal Kit (Gram-Positive Bacteria) (Illumina, MRZGP126). Illumina stranded whole transcriptome sequencing libraries were prepared using NEBNext Ultra Directional RNA library prep kit for Illumina (NEB, E7420S). The standard protocol for use with Ribosome Depleted RNA was followed except that, after second strand synthesis, the samples were precipitated with 1 µL (20 ng µL^−1^) glycogen and 1/10 vol. 3 M sodium acetate. Pellets were washed once with 80% ethanol, followed by 70% ethanol and after air-drying, pellets were resuspended in 58 µL of water. The standard protocol for use with Ribosome Depleted RNA was resumed for the remaining steps, except libraries were size selected using Agencourt AMPure XP beads at a 1.5 x ratio to retain the smaller sized fraction (~150 bp). The NEB Next Multiplex Oligos for Illumina kit (Primer set 1) (NEB, E7335S) was used to generate barcoded multiplex libraries. Library QC was performed using bioanalyser HS kit (Agilent biotechnologies, 5067–4626) and libraries were quantified using qPCR (Kapa Biosystems, KK4824). Libraries were pooled at desired concentrations, denatured and loaded for sequencing according to the manufacturer’s instructions. Sequencing was performed over 3 runs on the Illumina MiSeq sequencing platform to generate 2 × 75 bp reads.

For differential gene expression analysis the sequencing reads were mapped onto the annotated *C. difficile* strain 630 reference genome (http://www.ncbi.nlm.nih.gov/nuccore/115249003) with appropriate alignment software. The aligned files were then processed for tag counts per location mapped or normalised tag counts (RPKMs) and differential gene expression analysis. The DeepSeq Filtering Pipeline for Read Mapping was used to filter reads with low sequencing score, in addition to reads aligned to adaptor sequences. Reads from the sequencer were QC checked using FASTQC, then trimmed and filtered for low quality bases and adaptor sequences, and QC checked once more. Reads that passed this filter were mapped onto the reference genome in the context of known gene exon coordinates using the bwa mapping tool (http://bio-bwa.sourceforge.net/). Read alignments were recorded in a BAM formatted alignment file (named *.bam), and companion BAM index file (named *.bam.bai). Read alignments, both primary and unique, were then filtered further according to their mapping quality score (MAPQ). For gene expression, MAPQ20 uniquely aligned reads were used to generate counts per gene using ‘htseq-count’, which determines the number of uniquely aligned reads per gene (http://www-huber.embl.de/users/anders/HTSeq/doc/count.html).

These counts were used as the input for the DESeq program^84,85^. DESeq models the distribution of the counts data in each sample and then compares the distributions to determine differentially expressed genes, with significantly differentially expressed genes having an adjusted p value < 0.05. The program implements a single analytical approach and when RNA–seq samples with biological replicates are available, as is the case here, DESeq analyses the variance between them in order to better model the expression values of individual genes within the group of replicates.

The data discussed herein has been deposited in NCBI’s Gene Expression Omnibus^86^ repository (http://www.ncbi.nlm.nih.gov/geo/query/acc.cgi?acc=GSE112422) with accession number GSE112422.

### Data processing

Transcriptome sequence data was obtained from DeepSeq as a summary MS Excel file containing a list of genes with cognate base mean values for BHI medium (BHI, base mean A) and faecal water medium (FW, base mean B) growth conditions, in addition to p value, p-adjusted value and the ratio of FW/BHI base mean values, sorted by p-adjusted (padj) value from low to high. Some 1687 genes had p < 0.05, 1153 genes had padj < 0.05, reducing to 889 genes for which padj was < 0.001. The base mean values for these 889 genes were used to calculate log2 values for each FW/BHI ratio, from which was calculated the absolute fold–change for each gene. Subsequent analysis was undertaken with the statistically robust master list of 889 differentially expressed (DE) genes with pdaj < 0.001 and FC > 1.45. The NCBI *C. difficile* strain 630 genome (http://www.ncbi.nlm.nih.gov/nuccore/115249003) was used as a starting point for addition of the *C. difficile* strain 630 locus annotations^87,88^, in addition to protein name and Subtilist functional category^89,90^. This process was carried out essentially as in our previous work^45,46^ using the NCBI CDD database, BioCyc pathway tools and metacyc visual pathways software^82,91,92^ combined with literature searching to arrive at a functional role/categorisation and to identify predicted co–regulated genes and operon structures (for complete list of DE genes see Supplementary Table S3).

### Reverse transcription and qPCR

As in our previous work^44,45^ differential gene expression data was corroborated using qRT-PCR, on aliquots of the same RNA samples that were sent for sequencing. cDNA was prepared from 500 ng aliquots of the extracted RNA samples and 50 ng of random hexamer primer (Promega, WI, USA) with a SuperScript II Reverse Transcriptase kit (Invitrogen, Renfrewshire, UK). Successful reverse transcription and generation of cDNA was confirmed by PCR using *rpsJ*, *gyrA* or *adk* primers (Table 3), as compared to the “minus RT” controls. Quantitative PCR (qPCR) was performed on a LightCycler480 instrument using a Master SYBR Green 1 kit (Roche Diagnostics, UK). Standard curves were prepared by creating a 5–fold serial dilution (1, 1:5, 1:25, 1:125, 1:625, 1:3125, and 1:15125) of the pooled cDNA samples from all cultures with nuclease-free water. qPCR target run reactions were set up in technical triplicates. Bulk mastermix containing 5 µL of 2–fold concentrated master mix, 1 µL each of forward and reverse primer (at a concentration of 10 µM), 2 µL of nuclease free H_2_O and 1 µL of a 1–in–10 dilution of the relevant cDNA template was prepared and 10 µL aliquots of this added to the plate. qPCR cycling conditions comprised an initial denaturation stage of 95 °C for 5 min followed by 40 cycles of 95 °C for 10 s, 57 °C for 10 s and 72 °C for 10 s. Melting curve analysis of target runs, in addition to “no template” and “no reverse transcriptase” controls confirmed the specificity of amplification.

Roche Rel-Quant software (Roche Diagnostics, UK) was used to generate a C_q_ value for each sample using the second derivative maximum method. C_q_ values were transferred to Excel and the arithmetic mean of technical replicates was determined. These values were then log transformed to relative quantities (RQ) using the information gained from standard curves previously constructed for each primer pair, thus ensuring PCR efficiencies were calculated accurately for each gene. All target gene RQs were normalised against the geometric mean of the reference gene RQs by dividing the former by the later (target/housekeeping) to generate normalised relative quantity (NRQ) values. Control sample NRQ values (BHIS) were subsequently used as a calibrator and corrected to 1 with all experimental NRQ values (FW media) being expressed as a relative expression ratio to the calibrator for each gene. Values were subsequently expressed as fold change ratios relative to the BHI control (Supplementary Data File 1, Table S4).

### Liquid Chromatography-Mass Spectrometry (LC–MS) analysis of culture supernatant samples

#### *LC–MS*^*n*^*Analysis*

Culture media samples were frozen and transported to the Hutton Institute on dry ice where they were stored at −70 °C prior to analysis. After thawing on ice, samples (1 mL) were vortexed then transferred to 2 mL microcentrifuge tubes and centrifuged at 10,000 × g for 10 min at 5 °C in a refrigerated microfuge. A sub-sample (0.5 mL) was removed and placed in a 0.45 µm PTFE filter vial (Thomson Instrument Company, supplied by Bioprocess Engineering Services Ltd, Kent, UK) prior to analysis using the LTQ-Orbitrap XL LC−MS system. Samples were analysed using a LC system consisting of a quaternary pump (Thermo Fisher Scientific, Accella 600) and a PDA detector (Thermo Fisher Scientific, Accella) coupled to an LTQ Orbitrap XL mass spectrometer (Thermo Fisher Scientific, Hemel Hempstead, U.K.). Duplicate 10 μL samples were injected in part-loop mode onto a 2 × 150 mm (4 μm) Synergy Hydro-RP 80Ä column fitted with a C18 4 × 2 mm Security Guard cartridge (Phenomenex Ltd, Macclesfield, UK). Autosampler and column temperatures were maintained at 6 °C and 30 °C, respectively. The samples were analysed at a flow rate of 0.3 mL/min using a binary mobile phase of (A) 0.1% aqueous formic acid and (B) 0.1% formic acid in acetonitrile/water (1:1, vol/vol) with the following gradient: 0–4 min, 5% B; 4–22 min, 5–50% B; 22–32 min, 50–10 0% B. Following each analysis, the column was equilibrated for 5 min under starting solvent conditions. Mass detection was carried out using an LTQ Orbitrap XL mass spectrometer in negative ESI mode. Two scan events were employed; full-scan analysis (130–2000 *m/z*) in profile peak mode was followed by data–dependent MS/MS in centroid peak mode of the three most intense ions using a collision energy of 45 eV, activation Q of 0.25, trapping time 30 ms, and isolation width of 2 *m/z*. Full scan data were collected at a mass resolution of 30,000 (full width half maximum–FWHM –defined at *m/z* 400). Scan speeds of 0.1 s and 0.4 s were applied in the LTQ and FT-MS respectively. The Automatic Gain Control was set to 1 × 10^5^ and 5 × 10^5^ for the LTQ and FT-MS, respectively. Prior to the analytical run, the LTQ and FT-MS were tuned to optimise conditions for the detection of ions in the mid detection range of *m/z* 80–2000, as well as being calibrated with the manufacturer’s recomended calibration mixture. ESI conditions were as follows: spray voltage −3.5 kV (ESI-); sheath gas 60; aux gas 30; capillary voltage −35 V (ESI−); tube lens voltage −100 V (ESI-); capillary temperature 380 °C. For the first three min of analysis, the eluent flow was directed to waste. All predicted formula data presented are accurate at <2 ppm.

#### Raw LC–MS data processing

The raw LC–MS data files were first converted into an MZML centroid format using the Proteowizard MSConvert software package. Each MZML based three-dimensional data matrix (intensity × *m/z* × time) for each per sample was converted (or deconvoluted) into a vector of peak responses, where a peak response is defined as the sum of intensities over a window of specified mass and time range (e.g. *m/z* = 102.1 ± 0.01 and time = 130 ± 10 s) using the freely available XCMS software (http://masspec.scripps.edu/xcms/xcms.php). A full description of the data deconvolution method performed within XC–MS is available^93^. In the current work, the band width setting was adjusted from 10 to 20 to accommodate the wider peak widths that result from HPLC as compared to UHPLC. The XC–MS deconvolution produced an MS Excel based X by Y matrix output as peak areas for detected peaks.

#### Statistical analysis of LC–MS data

The data from XCMS was loaded into SIMCA-P 12.0.1.0 software (Umetrics, available at https://umetrics.com) and principal components analysis (PCA) was carried out. PCA, using univariate scaling, clearly showed that the FW samples separated from the BHI-only samples on score 1, which explained 52% of the variation of the dataset. The beginning and end samples were clearly separated in scores 3 and 4 of the PCA, which explained 10% and 5%, respectively, of the variation (Supplementary Data File 1, Fig. S1). Following this robust PCA, a further discriminant analysis (optimized partial least squares, OPLS-DA) was performed with two classifications (“start” and “end” of incubation), resulting in a model that described ~9% of the variation with a Q2 (cum) value of 0.851 (Supplementary Data File 1, Fig. S2).

Using the loadings plots from this OPLS–DA plot (Supplementary Data File 1, Fig. S3), the *m/z* signals that drove the separation for the “end of incubation” could be extracted into an Excel file (Supplementary Data File 1, Table S5). Returning to the original XCMS data, the abundance of these components before and after incubation (for both biological and technical replicates) was plotted as peak areas (Supplementary Data File 1, Figs S4, S5). Over 120 “potential up-at-end” components were selected by this process and the graphs were quality checked to select only those with a clear distinction between before and after peak areas (e. g. with no overlap between before and after replicates). A final step of manual peak checking was carried out to check MS peak quality and to exclude peaks of very low abundance which often yielded no MS^2^ data.

The XCMS data selected each *m/z* peak (along with any type of adduct ion(s) present) and the PutMedID set of workflows within the Taverna Workbench 1.7.2 software package^94^ was applied to predict putative metabolite identities using a library of known plant metabolites obtained from the Plant Metabolic Network PlantCyc database (http://www.plantcyc.org). In many cases, however, the putative identifications were not supported by subsequent examination (e. g.) of MS^2^ data. Therefore, further manual putative peak assignation was carried out by comparing the predicted molecular formulae and MS^2^ data with various databases and literature (Supplementary Data File 1, and Tables 1, 2).

### Equipment and settings

Images shown in Figs 1, 3, 4 and 5 were produced using MS Excel, individually exported in PDF and imported into GiMP 2.8 for construction and final labelling. MS traces comprising Fig. 2A,B were exported from the resident MS Xcalibur software (Thermo Fisher Scientific, Hemel Hempstead, U.K.) into a Word document. After conversion into Microsoft Office drawing objects, the traces were edited to incorporate peak labels in Microsoft Word, exported as.jpg files and imported into GiMP 2.8 for construction and final labelling of Fig. 2.

Images in Supplementary data file 1 (Figs S1, S3) were made using the graphics package inherent in SIMCA then copied into a Word document. Figs S4 and S5 were made using the graphic package in GENESTAT, then copied into a Word document. The MS traces comprising Fig. S6 were exported from the resident MS Xcalibur software (Thermo Fisher Scientific, Hemel Hempstead, U.K.) into a Word document. After conversion into Microsoft Office drawing objects, the traces were edited to incorporate peak labels in Microsoft Word. All Supplementary Figures S1–S6 were saved as a single pdf file.

### Ethical Approval

The Ulster University Research Ethics Committee exempted this study from review because donors were not involved in any intervention; the samples received were not collected by means of intervention and were used solely for preparation of a bacterial growth media. Written consent was obtained for provision of the donor faecal samples.

## Electronic supplementary material

Supplementary Data file 1

Supplementary Dataset Table S3

## Data Availability

All data generated or analysed during this study are included in this published article (and in Supplementary Information files). The RNAseq datasets generated and analysed in this work are available in the NCBI GEO repository (http://www.ncbi.nlm.nih.gov/geo/query/acc.cgi?acc=GSE112422) with accession number GSE112422.
