# Supplementary material for: Increased sporulation underpins adaptation of Clostridium difficile strain 630 to a biologically–relevant faecal environment, with implications for pathogenicity
Source: Sci Rep. 2018 Nov 12;8:16691. doi: 10.1038/s41598-018-35050-x (PMC6232153; doi:10.1038/s41598-018-35050-x)
Supplement: Supplementary file 1 — Supplementary Data file 1 [file 41598_2018_35050_MOESM1_ESM.pdf]

SUPPLEMENTARY DATA FILE 1

Increased sporulation underpins adaptation of *Clostridium difficile* strain 630 to a biologically–relevant faecal environment, with implications for pathogenicity

Nigel George Ternan[1]*, Nicola Diana Moore[1], Deborah Smyth[1], Gordon James McDougall[2], James William Allwood[2], Susan Verrall[2], Christopher Ian Richard Gill[1], James Stephen Gerard Dooley[1], Geoff McMullan[3].

ng.ternan@ulster.ac.uk; Moore-N18@email.ulster.ac.uk; debs.smyth@hotmail.co.uk; Gordon.McDougall@hutton.ac.uk; will.allwood@hutton.ac.uk; susan.verrall@hutton.ac.uk; c.gill@ulster.ac.uk; jsg.dooley@ulster.ac.uk; Geoff.McMullan@qub.ac.uk;

[1]Nutrition Innovation Centre for Food and Health (NICHE), School of Biomedical Sciences, University of Ulster, Coleraine, Co. Londonderry, N. Ireland, United Kingdom, BT52 1SA.

[2]Environmental and Biochemical Sciences Group, The James Hutton Institute, Invergowrie, Dundee, Scotland, United Kingdom, DD2 5DA.

[3]Institute for Global Food Security, School of Biological Sciences, Medical Biology Centre, Queen's University, Belfast, Northern Ireland, United Kingdom, BT9 7BL.

Contents

Table S1 – growth curve data	3
Table S2 – cell length data	4
Fig S1 PCA Analysis	5
Fig S2 OPLS-DA plot	6
Fig S3 Loadings plot from OPLS-DA analysis	7
Fig S4 Abundance plot example	8
Fig S5 “up at end” components (m/z values)	9
Fig S6 MS traces	18
Table S3 DEseq data	Separate, searchable, MS Excel file
Table S4 qRT-PCR data	19
Table S5 end of incubation signals	XX

Table S1

Figure 1a growth curve raw attenuance data

FW media Time (h)	Biol Rep 1 D650nm	Biol Rep 2 D650nm	Biol Rep 3 D650nm	Time (h)	Mean D650nm	st Dev
**0**	0.047	0.056	0.039	**0**	0.05	0.008505
**1**	0.056	0.021	0.036	**1**	0.04	0.017559
**2**	0.108	0.026	0.06	**2**	0.06	0.041199
**3**	0.255	0.096	0.14	**3**	0.16	0.0821
**4**	0.51	0.325	0.335	**4**	0.39	0.104043
**5**	0.75	0.53	0.62	**5**	0.63	0.110604
**6**	1.01	0.84	0.87	**6**	0.91	0.090738

BHIS control media Time (h)	Biol Rep 1 D650nm	Biol Rep 2 D650nm	Biol Rep 3 D650nm	Time (h)	Mean D650nm	st Dev
0	0.058	0.043	0.052	0	0.05	0.00755
1	0.08	0.051	0.07	1	0.07	0.014731
2	0.15	0.069	0.104	2	0.11	0.040624
3	0.295	0.146	0.199	3	0.21	0.075527
4	0.525	0.365	0.39	4	0.43	0.086072
5	0.915	0.575	0.635	5	0.71	0.181475
6	1.14	0.835	0.888	6	0.95	0.162961

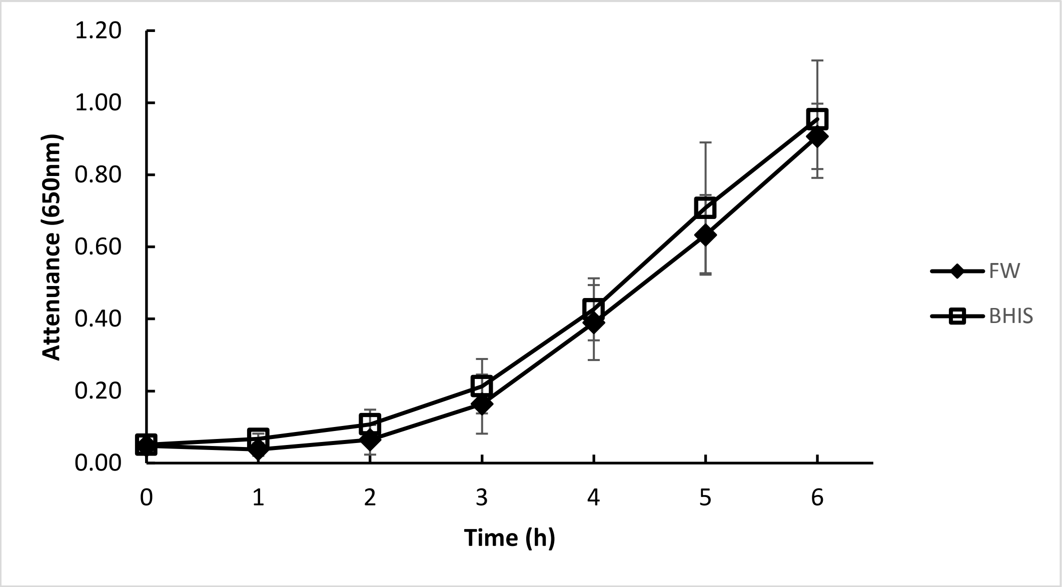

Table S2
(Figure 1b cell length data)

	Timepoint	Cell length (μm) FW	BHIS
Biol Rep 1 (100 cells)	0	3.34	3.72
Biol Rep 2 (100 cells)	0	3.28	3.84
Biol Rep 3 (100 cells)	0	3.06	3.66
Biol Rep 1 (100 cells)	1	3.64	4.45
Biol Rep 2 (100 cells)	1	3.91	4.1
Biol Rep 3 (100 cells)	1	2.99	4.6
Biol Rep 1 (100 cells)	2	3.95	4.6
Biol Rep 2 (100 cells)	2	3.84	4.01
Biol Rep 3 (100 cells)	2	3.00	4.37
Biol Rep 1 (100 cells)	3	4.21	4.84
Biol Rep 2 (100 cells)	3	3.81	3.66
Biol Rep 3 (100 cells)	3	3.52	4.21
Biol Rep 1 (100 cells)	4	4.4	3.96
Biol Rep 2 (100 cells)	4	3.88	2.96
Biol Rep 3 (100 cells)	4	4.87	3.57
Biol Rep 1 (100 cells)	5	4.47	3.78
Biol Rep 2 (100 cells)	5	4.41	3.31
Biol Rep 3 (100 cells)	5	4.48	3.07
Biol Rep 1 (100 cells)	6	4.34	3.45
Biol Rep 2 (100 cells)	6	3.97	3.25
Biol Rep 3 (100 cells)	6	4.64	3.08

Timepoint	FW Mean length (μm)	SD		BHIS Mean	SD
0	3.2	0.1	0	3.7	0.1
1	3.5	0.3	1	4.4	0.3
2	3.6	0.3	2	4.3	0.3
3	3.8	0.6	3	4.2	0.6
4	4.4	0.5	4	3.5	0.5
5	4.5	0.4	5	3.4	0.4
6	4.3	0.2	6	3.3	0.2

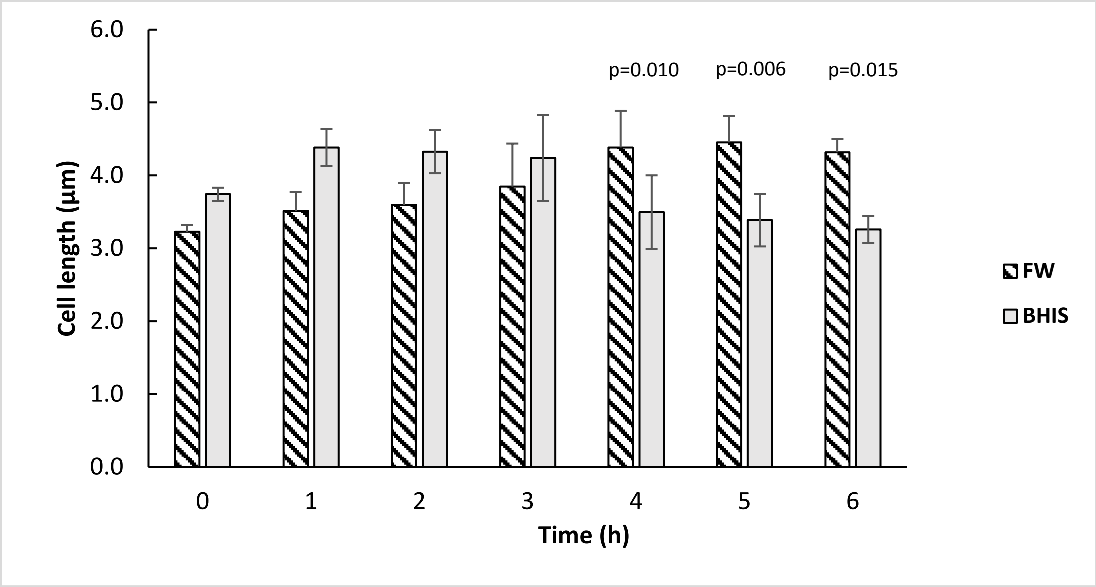

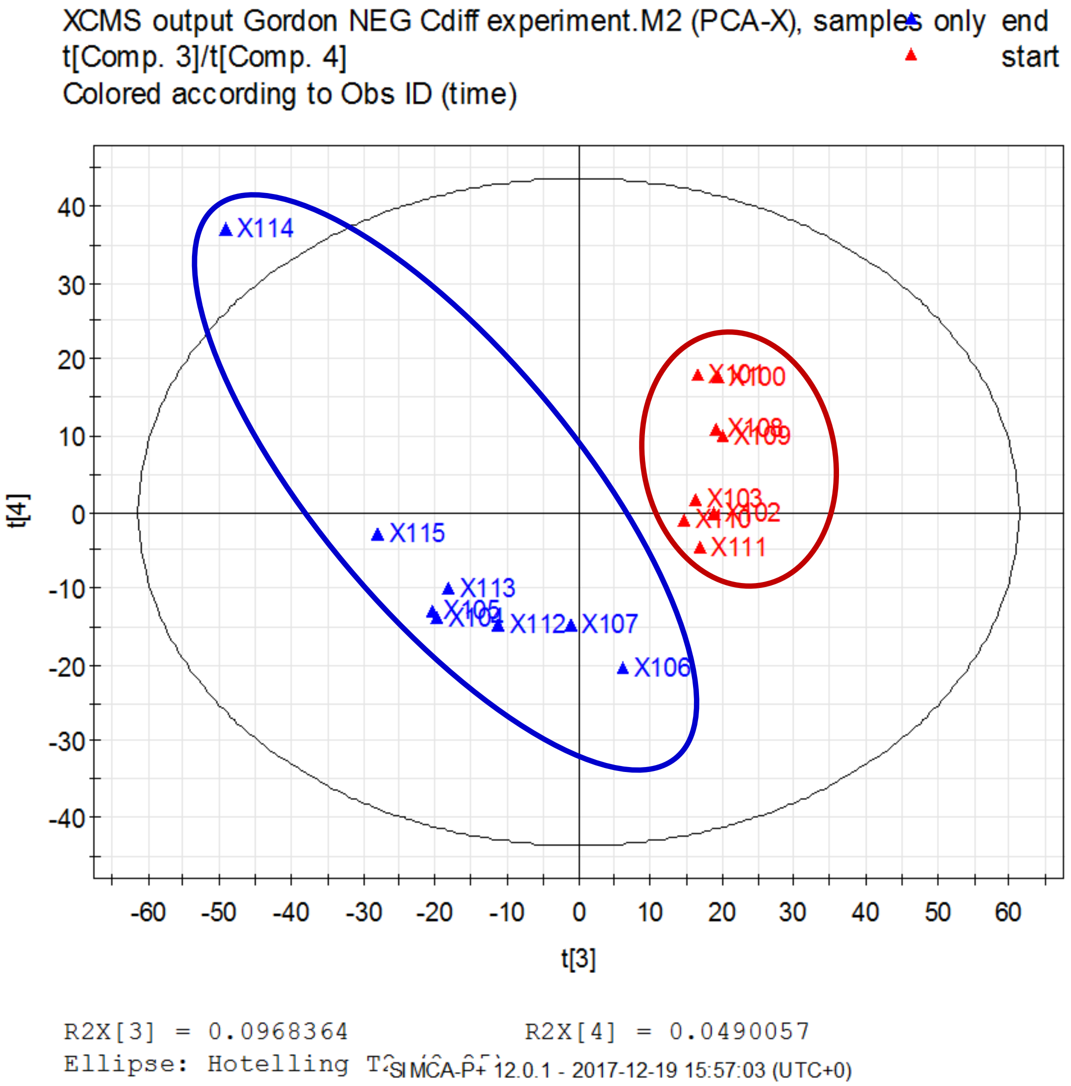

Fig. S1. Principal Components Analysis (PCA) of LCMS data

Blue = end of incubation samples

Red = start of incubation samples

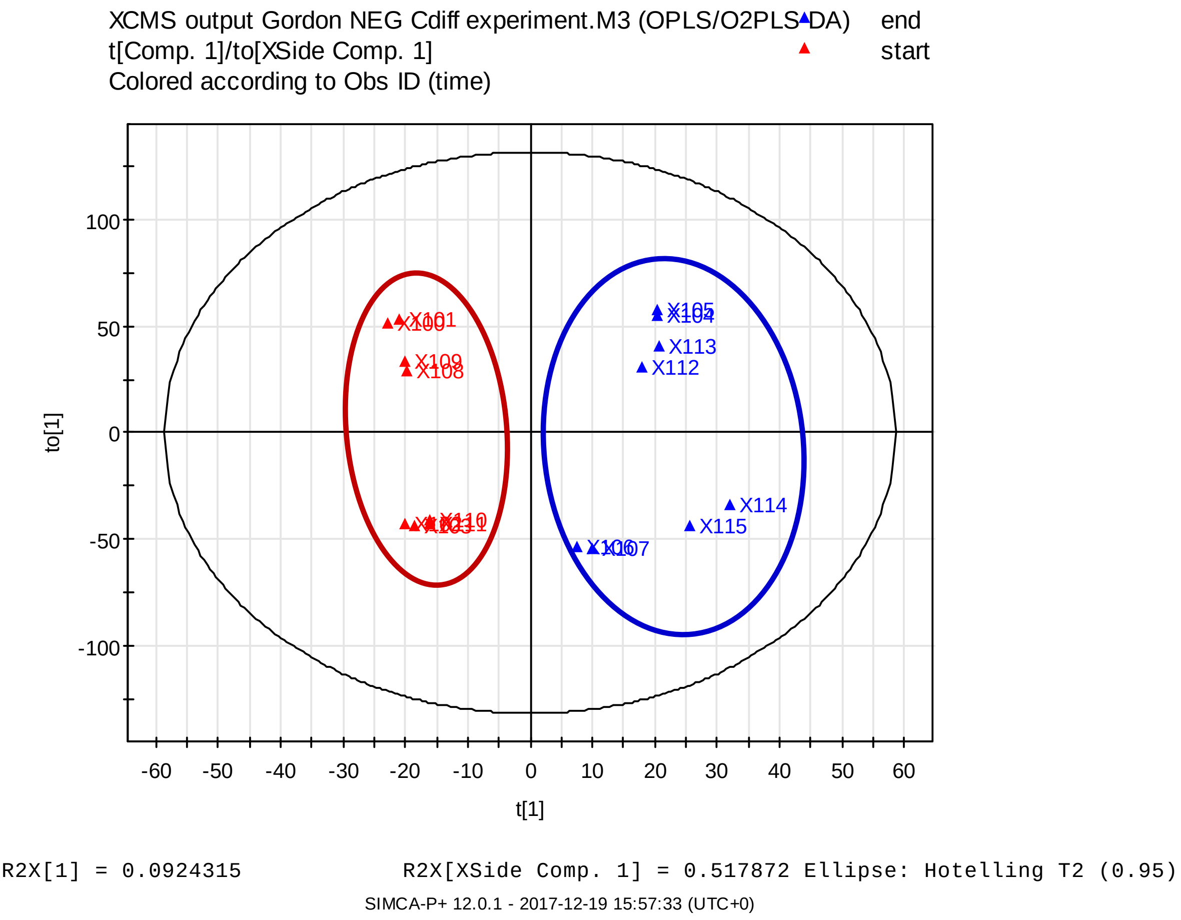

Fig. S2. OPLS-DA plot of LCMS data

Blue = end of incubation samples

Red = start of incubation samples

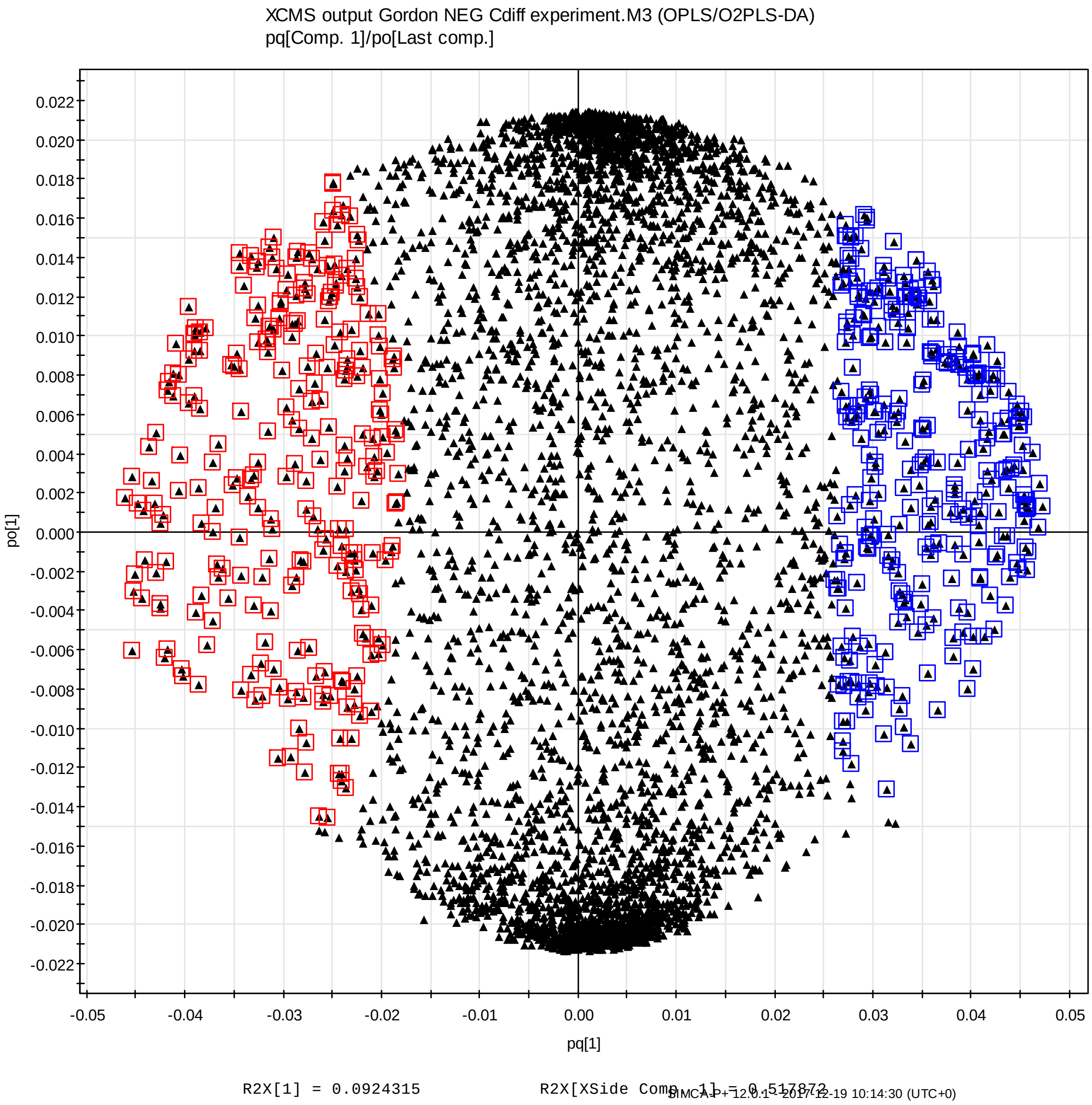

Fig. S3. Loadings plot from OPLS-DA analysis

Blue = m/z signals that contribute to separation of "end of incubation" samples

Red = m/z signals that contribute to separation of "start of incubation" samples

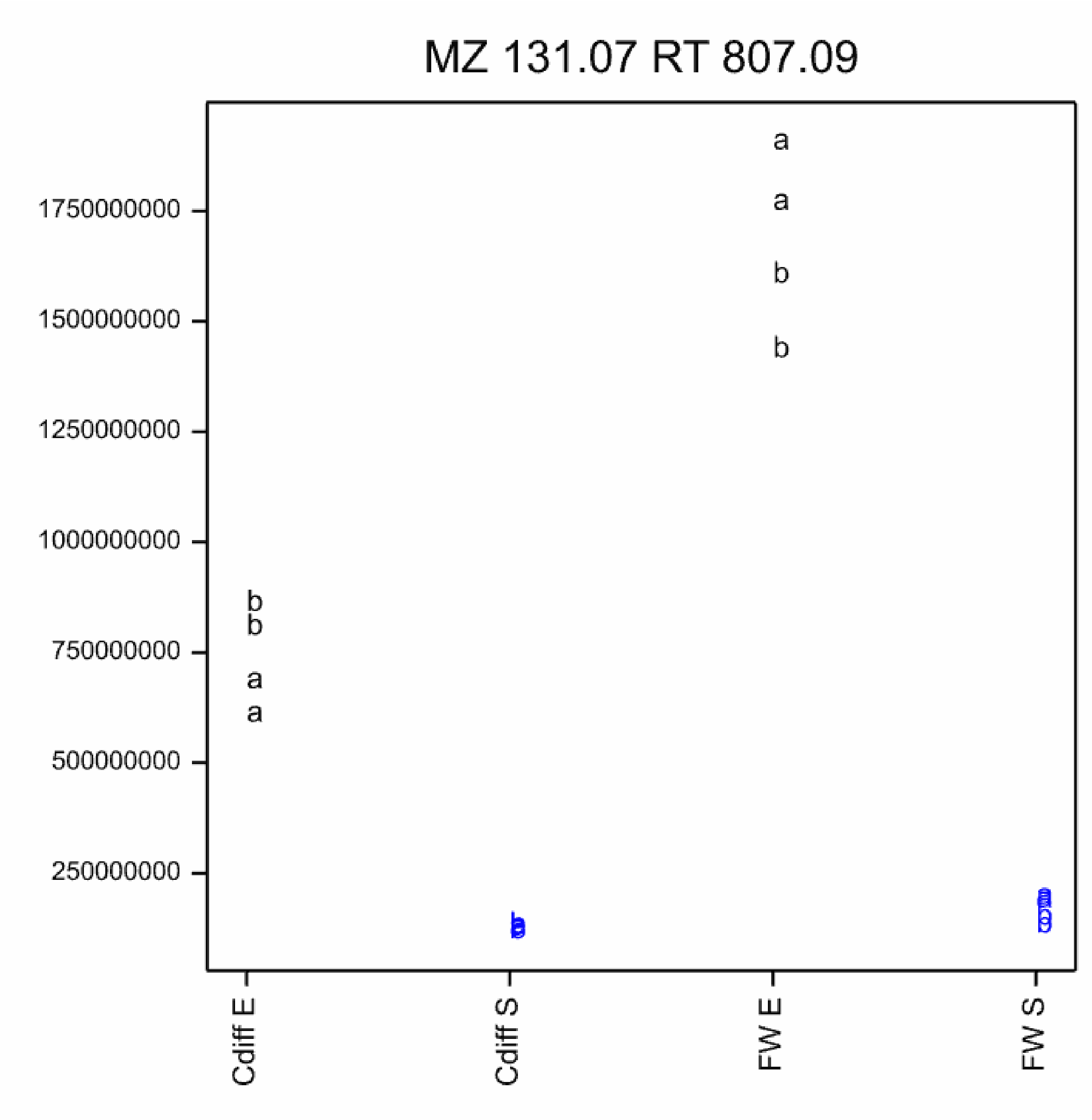

Fig. S4. Abundance plot for one putative "increased at end" component

Values denoted "a" and "b" are different biological replicates and are repeated as technical replicates. Blue values are the start peak areas.

Levels of this component are higher in the FW media at end of incubation.

Following 8 pages:

Fig. S5. Components (m/z values) identified as "up-at-end" by user identification and PCA analysis

Green = peaks identified by PCA analysis

BLUE & ORANGE = peaks identified by user examination

Dashed line = +1 isotope

Dotted line = +formic acid adduct or *m/z* 2 [M-H]-

S = start, E = end of incubation, a and b are biological replicates

Information on the identification of these "up-at-end" peaks is presented in Table 2.

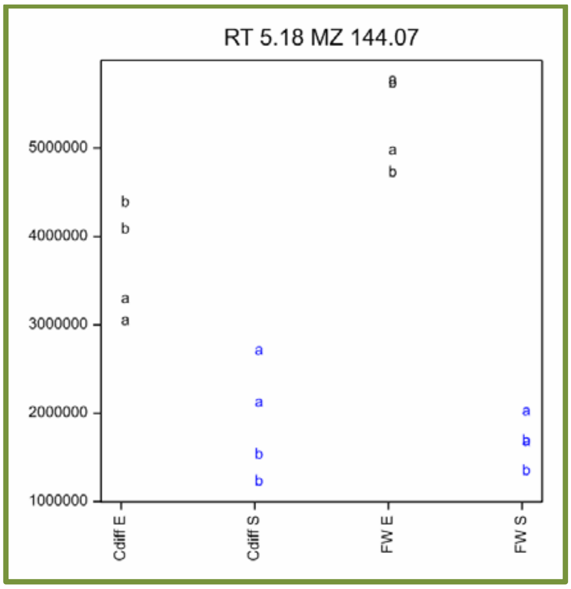
RT 5.18 MZ 144.07
5000000
4000000
3000000
2000000
1000000
Cdiff E
Cdiff S
FW E
FW S

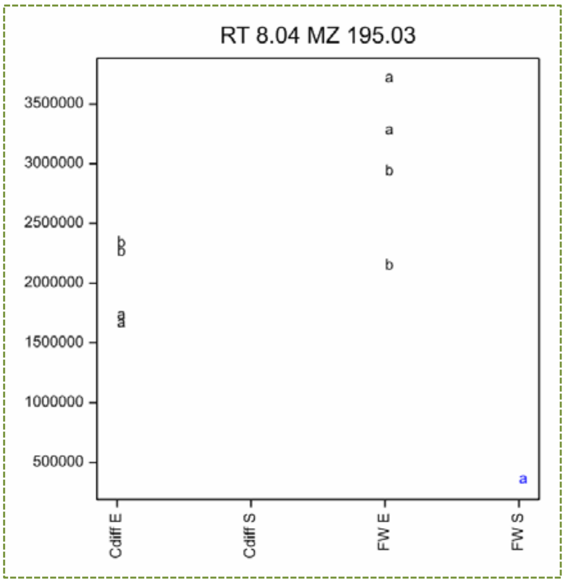
RT 8.04 MZ 195.03
3500000
3000000
2500000
2000000
1500000
1000000
500000
Cdiff E
Cdiff S
FW E
FW S

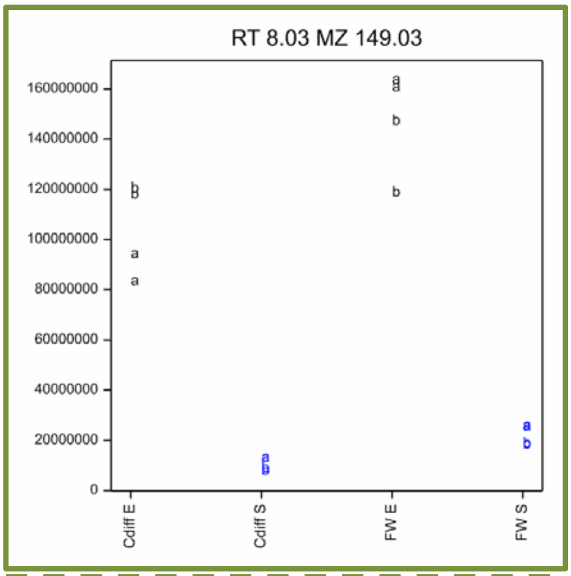
RT 8.03 MZ 149.03
160000000
140000000
120000000
100000000
80000000
60000000
40000000
20000000
0
Cdiff E
Cdiff S
FW E
FW S

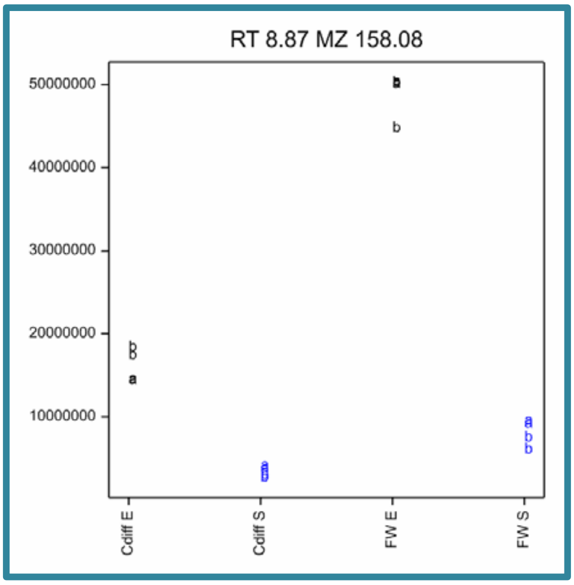
RT 8.87 MZ 158.08
50000000
40000000
30000000
20000000
10000000
Cdiff E
Cdiff S
FW E
FW S

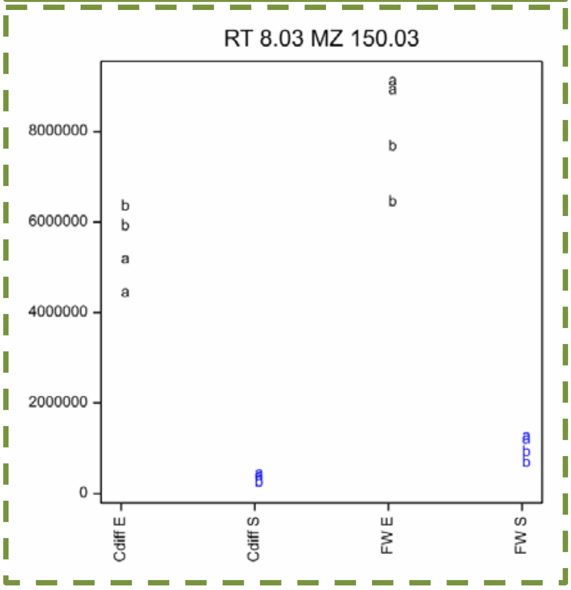
RT 8.03 MZ 150.03
8000000
6000000
4000000
2000000
0
Cdiff E
Cdiff S
FW E
FW S

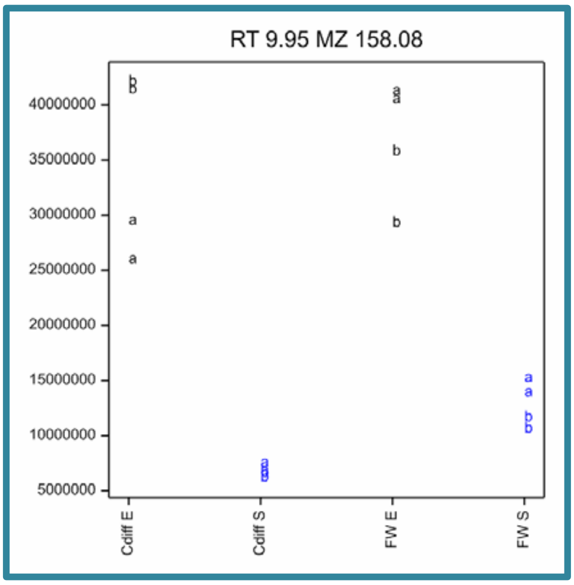
RT 9.95 MZ 158.08
40000000
35000000
30000000
25000000
20000000
15000000
10000000
5000000
Cdiff E
Cdiff S
FW E
FW S

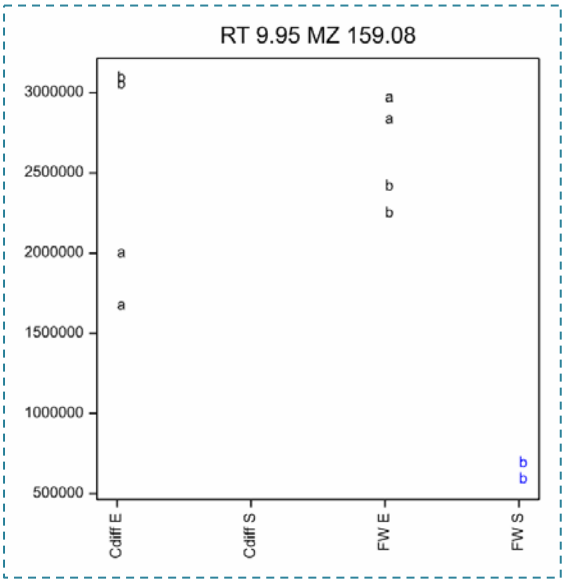
RT 9.95 MZ 159.08
3000000
2500000
2000000
1500000
1000000
500000
Cdiff E
Cdiff S
FW E
FW S

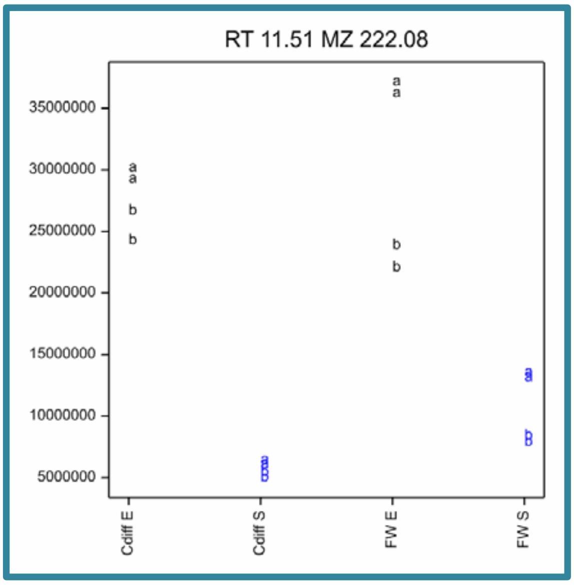
RT 11.51 MZ 222.08
35000000
30000000
25000000
20000000
15000000
10000000
5000000
Cdiff E
Cdiff S
FW E
FW S

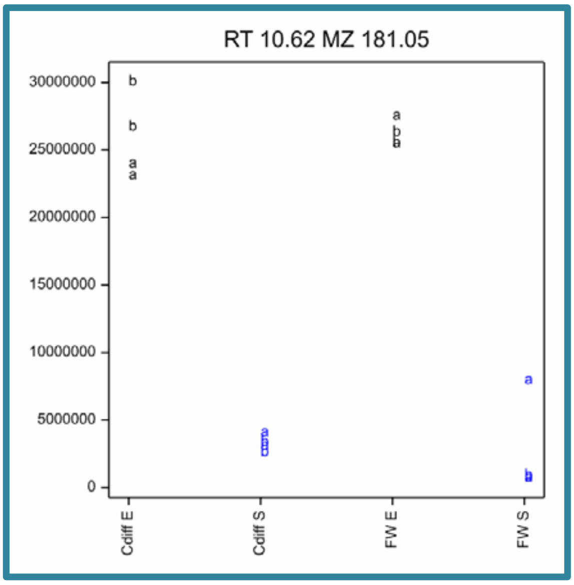
RT 10.62 MZ 181.05
30000000
25000000
20000000
15000000
10000000
5000000
0
Cdiff E
Cdiff S
FW E
FW S

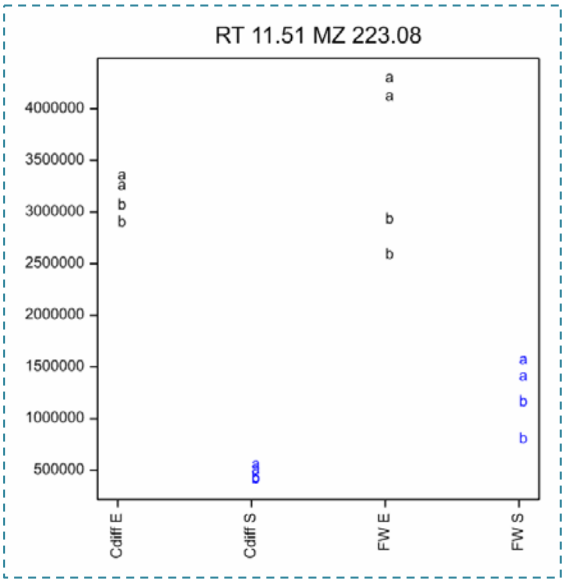
RT 11.51 MZ 223.08
4000000
3500000
3000000
2500000
2000000
1500000
1000000
500000
Cdiff E
Cdiff S
FW E
FW S

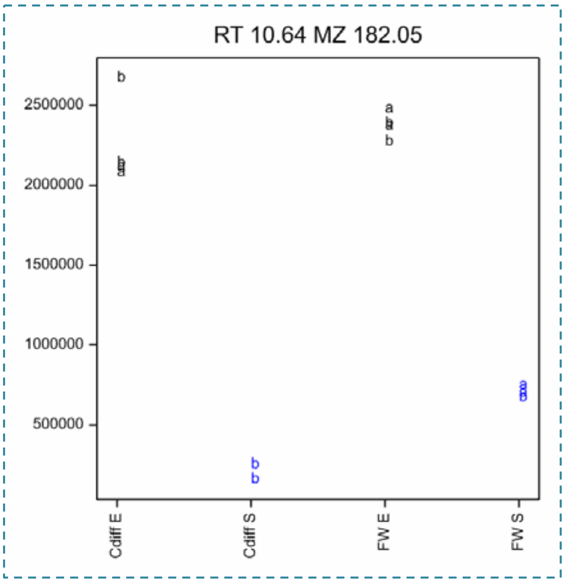
RT 10.64 MZ 182.05
2500000
2000000
1500000
1000000
500000
Cdiff E
Cdiff S
FW E
FW S

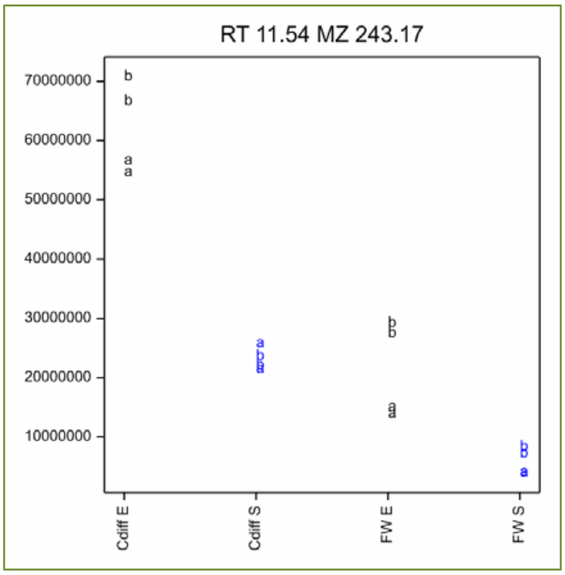
RT 11.54 MZ 243.17
70000000
60000000
50000000
40000000
30000000
20000000
10000000
Cdiff E
Cdiff S
FW E
FW S

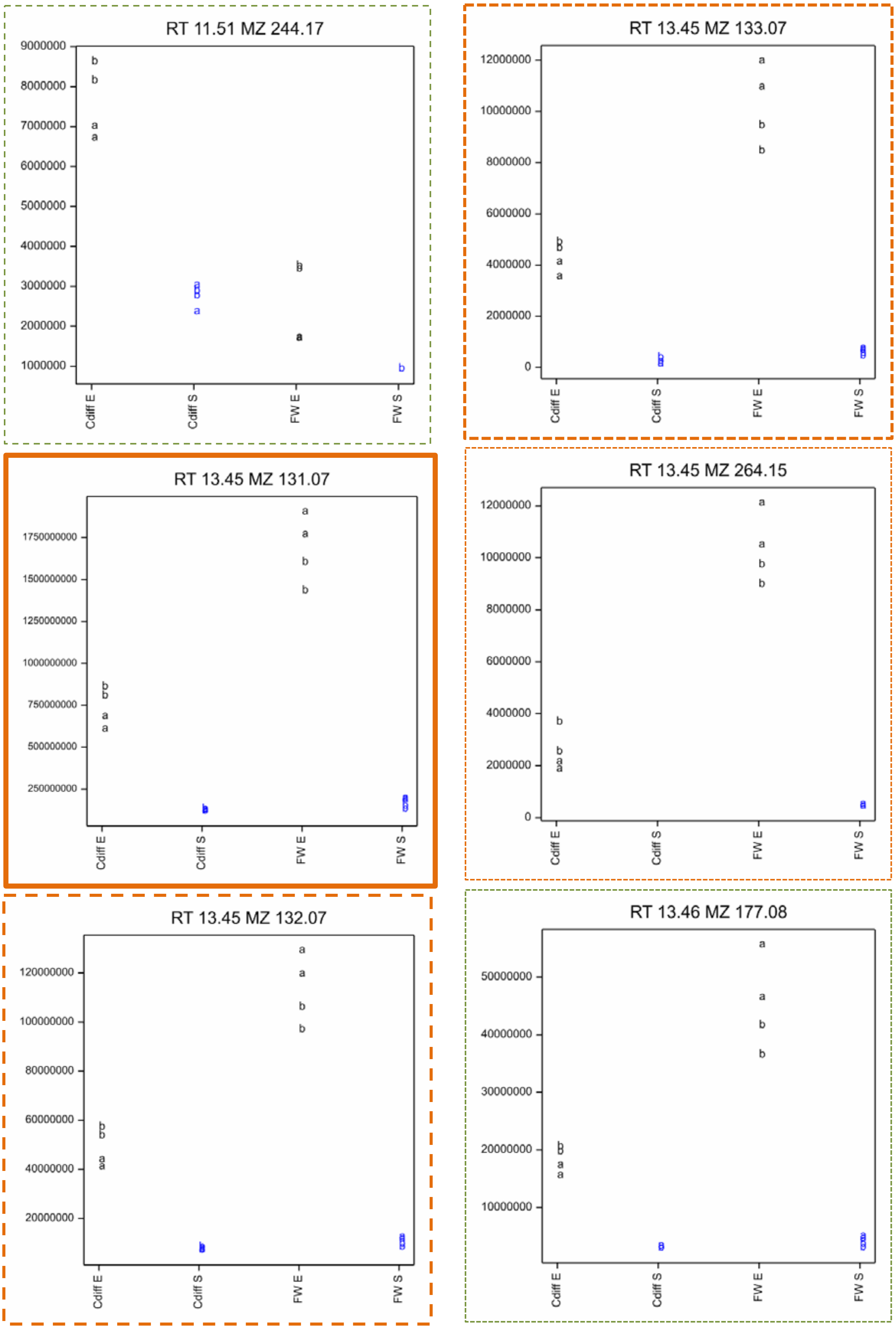

RT 11.51 MZ 244.17
9000000
8000000
7000000
6000000
5000000
4000000
3000000
2000000
1000000
Cdiff E
Cdiff S
FW E
FW S
RT 13.45 MZ 133.07
12000000
10000000
8000000
6000000
4000000
2000000
0
Cdiff E
Cdiff S
FW E
FW S
RT 13.45 MZ 131.07
1750000000
1500000000
1250000000
1000000000
750000000
500000000
250000000
Cdiff E
Cdiff S
FW E
FW S
RT 13.45 MZ 264.15
12000000
10000000
8000000
6000000
4000000
2000000
0
Cdiff E
Cdiff S
FW E
FW S
RT 13.45 MZ 132.07
120000000
100000000
80000000
60000000
40000000
20000000
Cdiff E
Cdiff S
FW E
FW S
RT 13.46 MZ 177.08
50000000
40000000
30000000
20000000
10000000
Cdiff E
Cdiff S
FW E
FW S

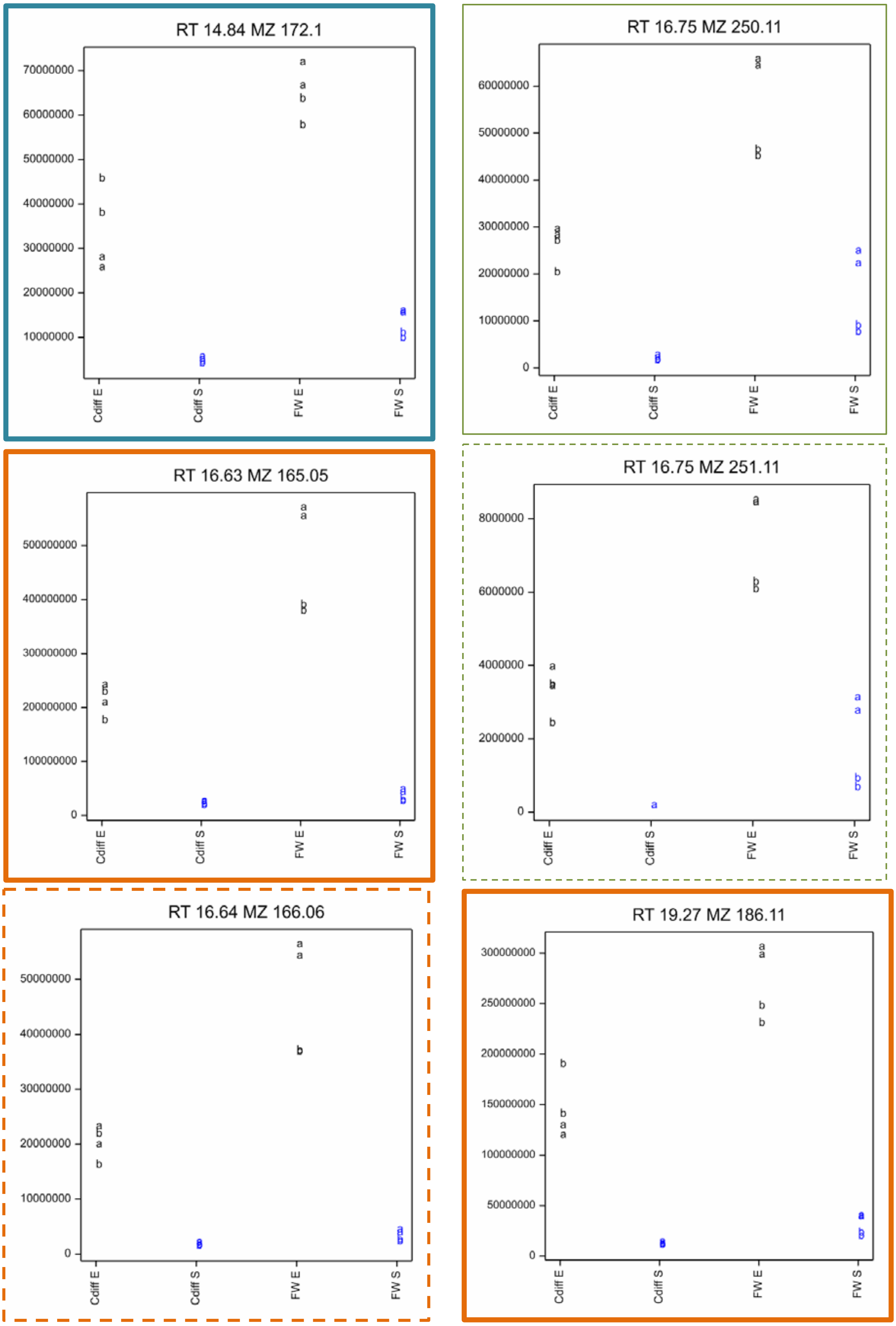
RT 14.84 MZ 172.1
70000000
60000000
50000000
40000000
30000000
20000000
10000000
Cdiff E
Cdiff S
FW E
FW S
RT 16.75 MZ 250.11
60000000
50000000
40000000
30000000
20000000
10000000
0
RT 16.63 MZ 165.05
500000000
400000000
300000000
200000000
100000000
0
RT 16.75 MZ 251.11
8000000
6000000
4000000
2000000
0
RT 16.64 MZ 166.06
50000000
40000000
30000000
20000000
10000000
0
RT 19.27 MZ 186.11
300000000
250000000
200000000
150000000
100000000
50000000
0

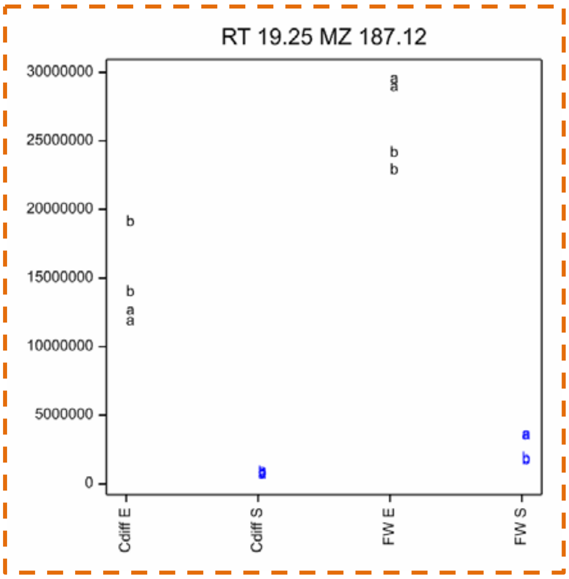
RT 19.25 MZ 187.12
30000000
25000000
20000000
15000000
10000000
5000000
0
Cdiff E
Cdiff S
FW E
FW S

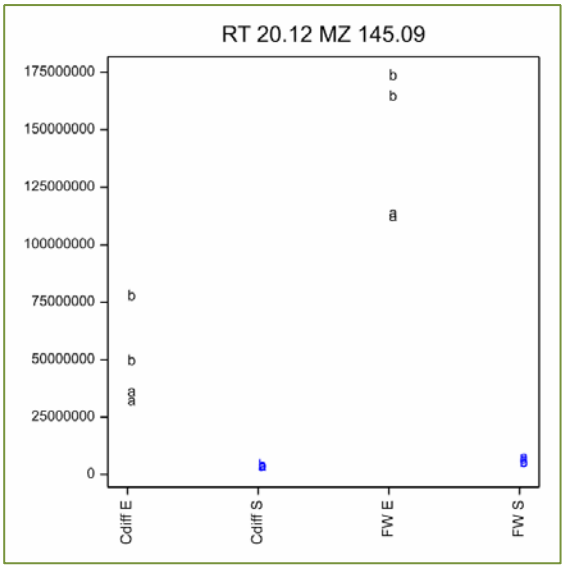
RT 20.12 MZ 145.09
175000000
150000000
125000000
100000000
75000000
50000000
25000000
0
Cdiff E
Cdiff S
FW E
FW S

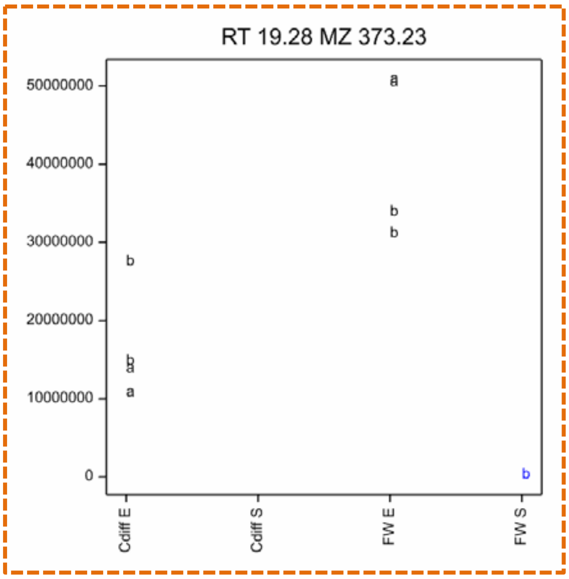
RT 19.28 MZ 373.23
50000000
40000000
30000000
20000000
10000000
0
Cdiff E
Cdiff S
FW E
FW S

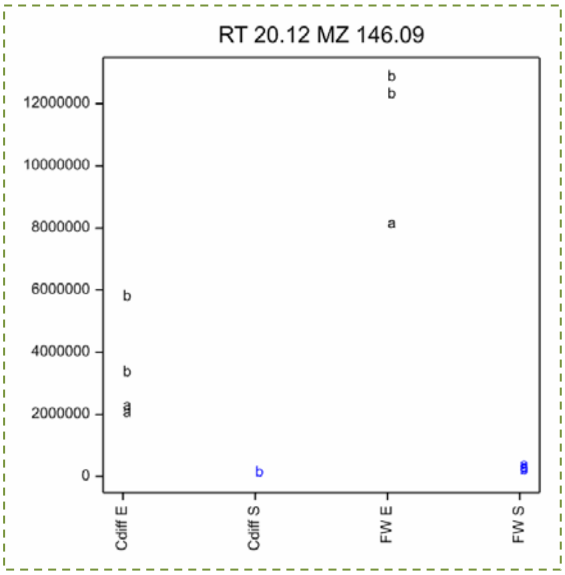
RT 20.12 MZ 146.09
12000000
10000000
8000000
6000000
4000000
2000000
0
Cdiff E
Cdiff S
FW E
FW S

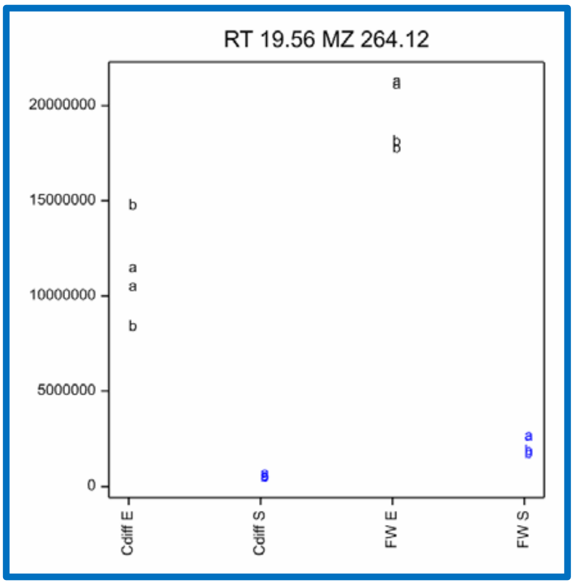
RT 19.56 MZ 264.12
20000000
15000000
10000000
5000000
0
Cdiff E
Cdiff S
FW E
FW S

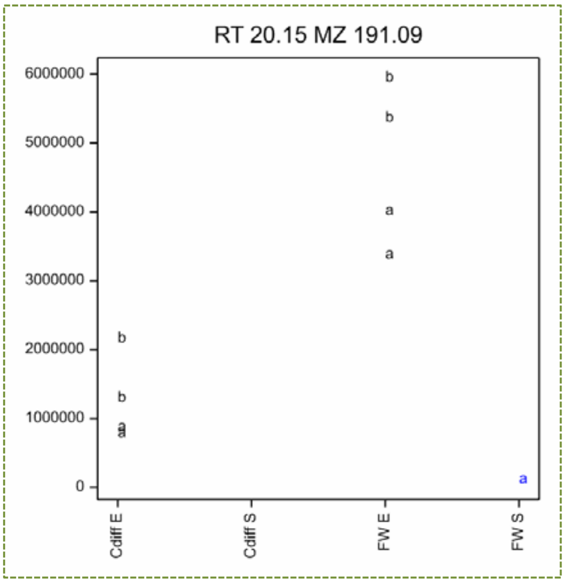
RT 20.15 MZ 191.09
6000000
5000000
4000000
3000000
2000000
1000000
0
Cdiff E
Cdiff S
FW E
FW S

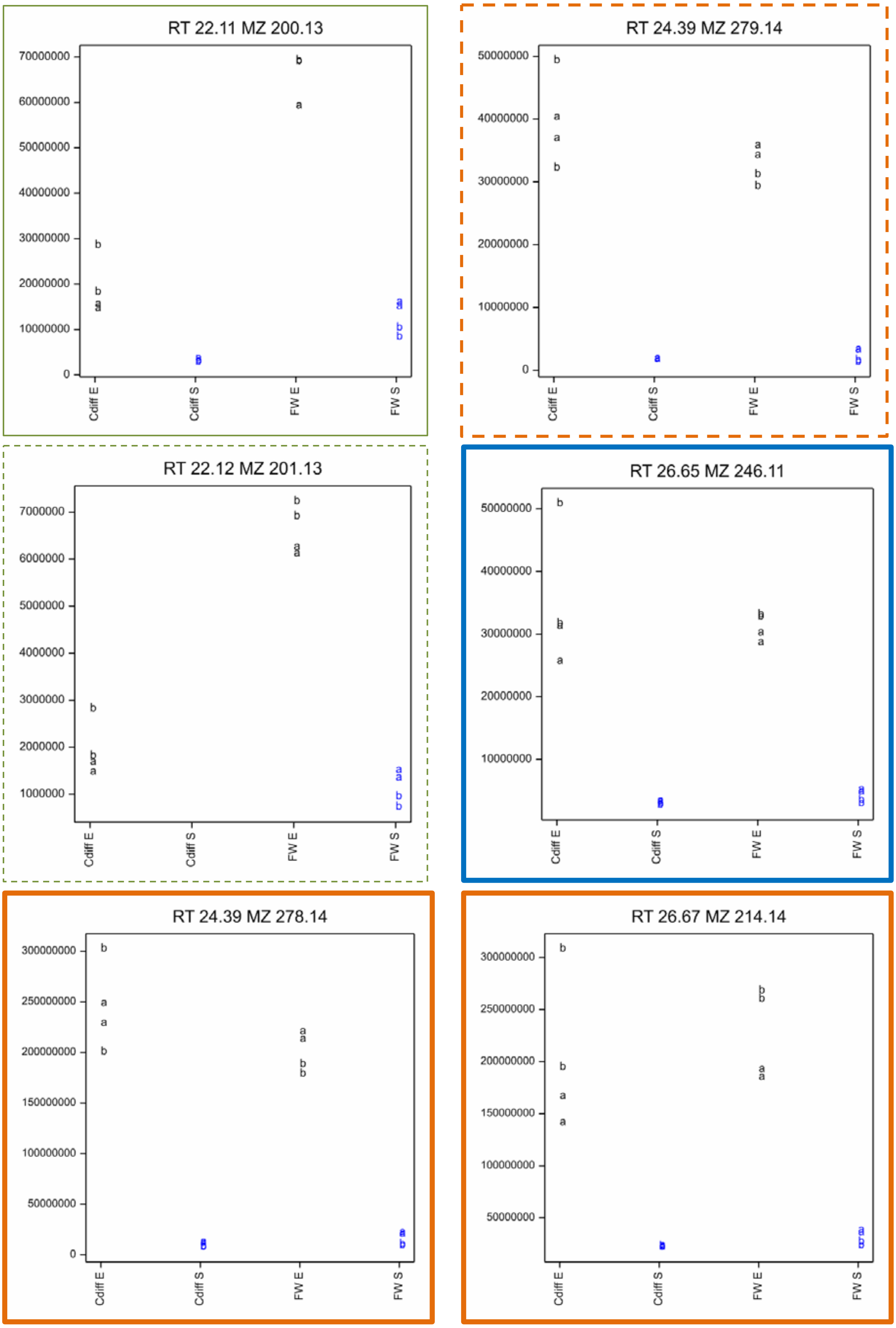
RT 22.11 MZ 200.13
RT 24.39 MZ 279.14
RT 22.12 MZ 201.13
RT 26.65 MZ 246.11
RT 24.39 MZ 278.14
RT 26.67 MZ 214.14
Cdiff E
Cdiff S
FW E
FW S

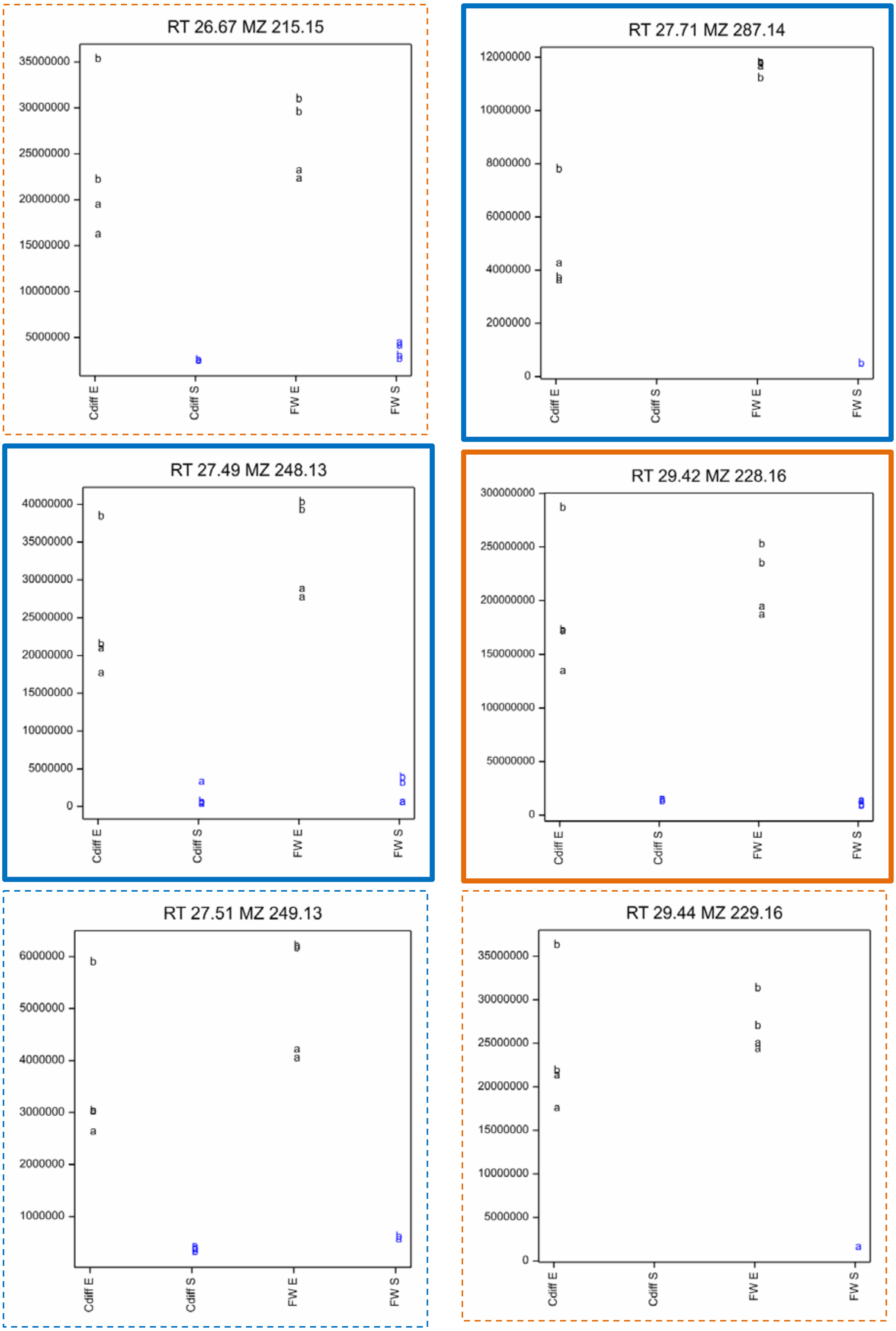
RT 26.67 MZ 215.15
RT 27.71 MZ 287.14
RT 27.49 MZ 248.13
RT 29.42 MZ 228.16
RT 27.51 MZ 249.13
RT 29.44 MZ 229.16
Cdiff E
Cdiff S
FW E
FW S

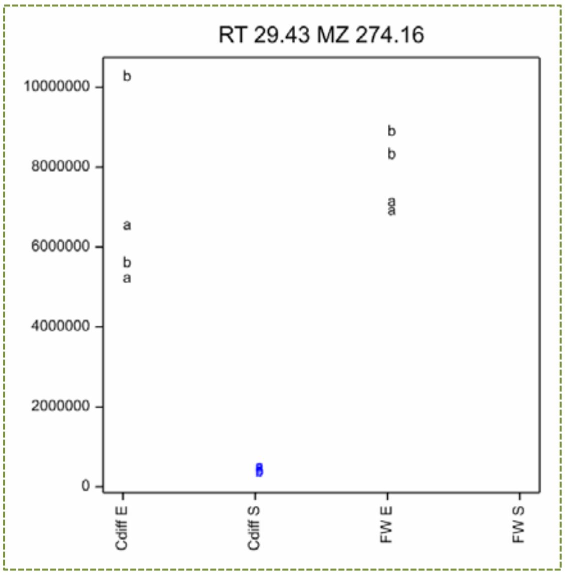
RT 29.43 MZ 274.16
10000000
8000000
6000000
4000000
2000000
0
Cdiff E
Cdiff S
FW E
FW S

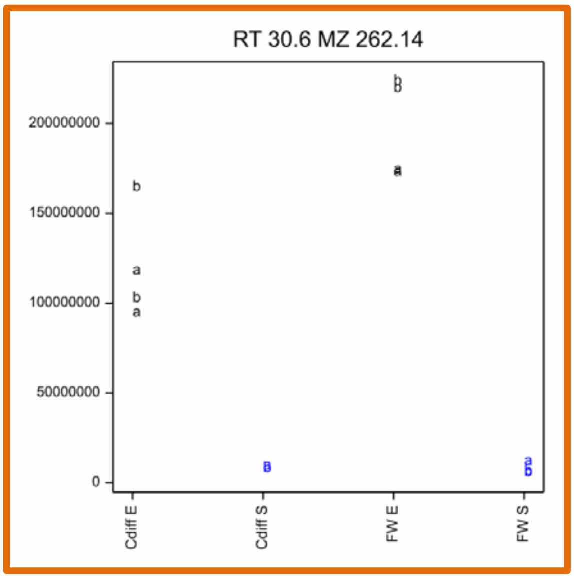
RT 30.6 MZ 262.14
200000000
150000000
100000000
50000000
0
Cdiff E
Cdiff S
FW E
FW S

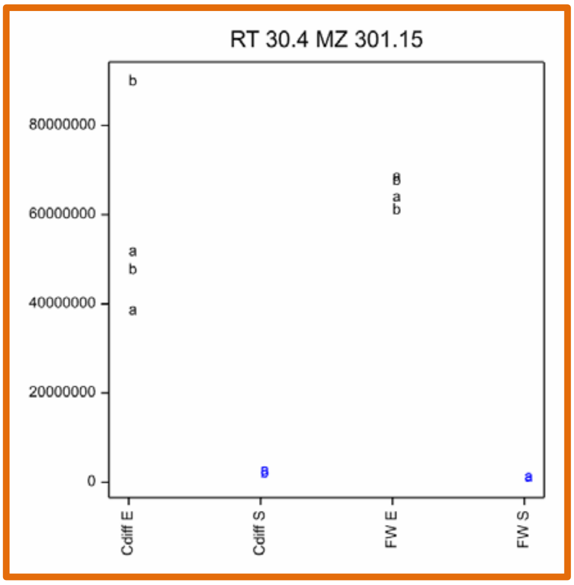
RT 30.4 MZ 301.15
80000000
60000000
40000000
20000000
0
Cdiff E
Cdiff S
FW E
FW S

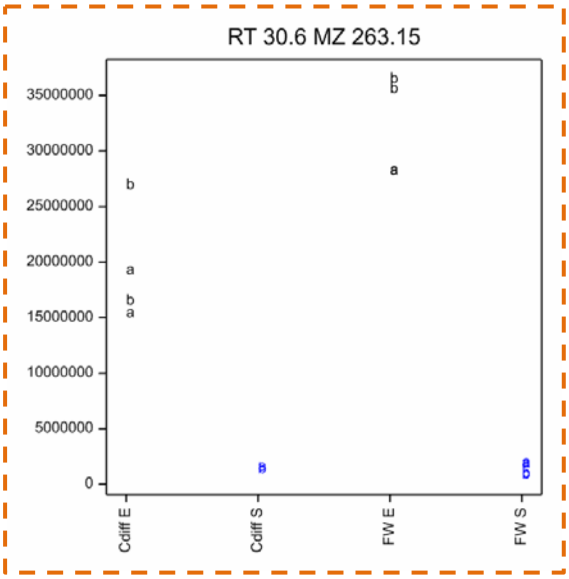
RT 30.6 MZ 263.15
35000000
30000000
25000000
20000000
15000000
10000000
5000000
0
Cdiff E
Cdiff S
FW E
FW S

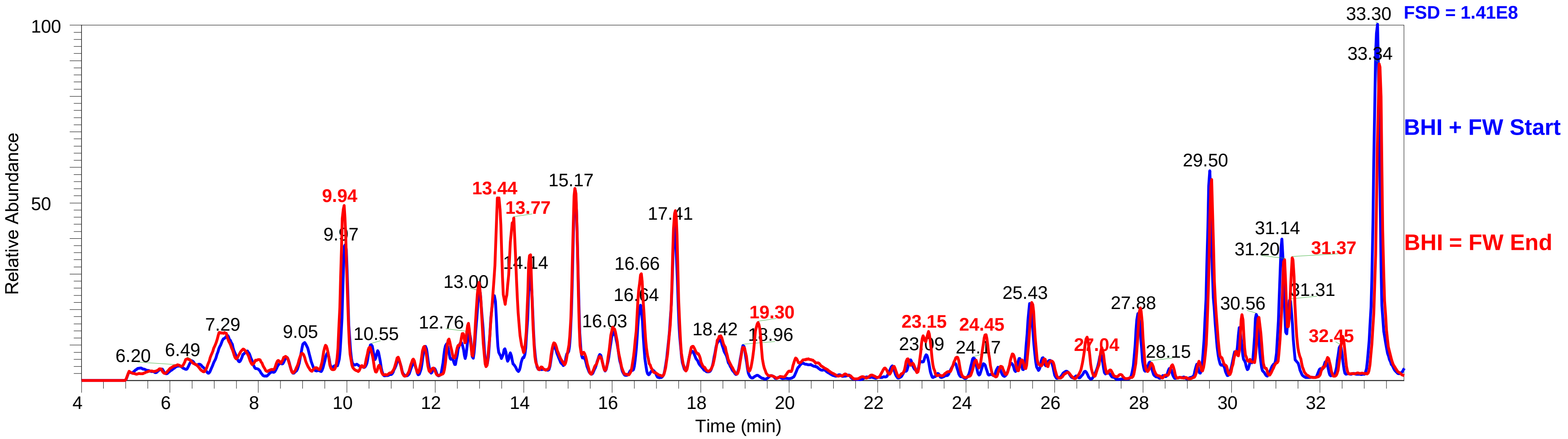

Fig. S6. Overlay of BHI + FW t=0 and BHI + FW end showing the peaks that increased during the growth of *C. difficile*.

The abundance of the FW derived peaks are largely retained

Table S4 **qRT-PCr data**

Reference Genes									
	**rpsJ**						**Gene**	**slope**	**y-intercept**
	FW 1	FW 2	FW 3	PBS 1	PBS 2	PBS 3	rpsJ	-3.432	18.29
#1	18.21	18.3	18.61	19.52	23.3	19.35			
#2	17.89	18.32	18.67	18.88	23.42	19.44			
#3	17.57	18.26	18.59	19.67	23.3	19.44			
Avera	17.89	18.293333	18.62	19.357	23.34	19.41			
Eq. lin	0.1165501	-0.000971	-0.097	-0.311	-1.47145	-0.32634			
RQ	1.3078265	0.9977661	0.8	0.4889	0.033772	0.471693			

	**gyrA**						**Gene**	**slope**	**y-intercept**
	FW 1	FW 2	FW 3	PBS 1	PBS 2	PBS 3	gyrA	-3.337	21.96
#1	20.54	20.53	21.33	23.2	26.08	21.95			
#2	20.03	20.45	21.25	22.24	26.12	21.86			
#3	20.11	20.3	21.27	22.2	26.01	21.88			
Avera	20.226667	20.426667	21.28	22.547	26.07	21.89667			
Eq. lin	0.5194286	0.4594946	0.203	-0.176	-1.23165	0.018979			
RQ	3.3069576	2.8806769	1.595	0.6671	0.058662	1.04467			

	**adK**						**Gene**	**slope**	**y-intercept**
	FW 1	FW 2	FW 3	PBS 1	PBS 2	PBS 3	adK	-3.486	21.95
#1	29.75	29.63	27.18	30.6	32.76	27.17			
#2	28.76	29.56	26.98	30.69	33.01	26.69			
#3	27.34	28.57	26.71	30.49	31.87	26.48			
Avera	28.616667	29.253333	26.96	30.593	32.54667	26.78			
Eq. lin	-1.912412	-2.095047	-1.436	-2.479	-3.03978	-1.38554			
RQ	0.0122346	0.0080344	0.037	0.0033	0.000912	0.041158			

Genes of Interest

	GroEL						Gene	slope	y-intercept
	FW 1	FW 2	FW 3	PBS 1	PBS 2	PBS 3	GroEL	-3.383	18.35
#1	21.38	21.49	19.99	23.2	23.73	18.44			
#2	20.7	21.18	19.62	22.96	23.46	19.22			
#3	19.97	20.74	19.37	22.82	23.36	18.99			
Average	20.68333	21.13667	19.66	22.99333	23.51667	18.88333			
Eq. line	-0.68972	-0.82373	-0.38723	-1.37255	-1.52724	-0.15765			
RQ	0.204304	0.150063	0.409987	0.042408	0.0297	0.695583			

	GroES						Gene	slope	y-intercept
	FW 1	FW 2	FW 3	PBS 1	PBS 2	PBS 3	GroES	-3.352	18.98
#1	19.75	19.48	18.05	20.9	22.79	17.85			
#2	19.21	19.44	17.99	20.94	22.8	17.88			
#3	18.88	19.28	17.98	20.98	22.8	17.88			
Average	19.28	19.4	18.00667	20.94	22.79667	17.87			
Eq. line	-0.0895	-0.1253	0.290374	-0.58473	-1.13862	0.331146			
RQ	0.813769	0.749379	1.951524	0.26018	0.072674	2.143609			

	fliC						Gene	slope	y-intercept
	FW 1	FW 2	FW 3	PBS 1	PBS 2	PBS 3	fliC	-3.456	19.24
#1	20.13	20.61	19.96	19.33	20.6	19.05			
#2	19.96	20.22	20.07	19.16	20.58	19			
#3	19.8	20.2	20.05	19.26	20.59	18.95			
Average	19.96333	20.34333	20.02667	19.25	20.59	19			
Eq. line	-0.2093	-0.31925	-0.22762	-0.00289	-0.39063	0.069444			
RQ	0.617593	0.479456	0.592075	0.99336	0.406794	1.173396			

	fliD						Gene	slope	y-intercept
	FW 1	FW 2	FW 3	PBS 1	PBS 2	PBS 3	fliD	-3.426	22.88
#1	24.26	24.52	24.8	23.62	25.62	23.73			
#2	24.45	24.27	24.07	23.48	25.29	23.65			
#3	23.48	24.19	24.09	23.49	25.31	23.45			
Average	24.06333	24.32667	24.32	23.53	25.40667	23.61			
Eq. line	-0.3454	-0.42226	-0.42032	-0.18973	-0.7375	-0.21308			
RQ	0.451442	0.378215	0.379914	0.646062	0.183022	0.612243			

Genes of Interest									
	tcdA						Gene	slope	y-intercept
	FW 1	FW 2	FW 3	PBS 1	PBS 2	PBS 3	tcdA	-3.418	24.26
#1	29.77	29.44	29.43	26.99	27.93	27.26			
#2	30.45	29.02	28.47	26.91	27.88	27.11			
#3	28.66	28.92	28.48	26.92	27.85	26.93			
Avera	29.626667	29.126667	28.79	26.94	27.88667	27.1			
Eq. lin	-1.570119	-1.423835	-1.326	-0.784	-1.06105	-0.8309			
RQ	0.026908	0.0376847	0.047	0.1644	0.086886	0.147606			

	SpoVB						Gene	slope	y-intercept
	FW 1	FW 2	FW 3	PBS 1	PBS 2	PBS 3	SpoVB	-3.308	28.62
#1	32.3	32.64	32.2	34.11	37.06	32.55			
#2	31.99	31.79	30.84	33.3	35.74	31.53			
#3	31.52	30.89	29.97	32.61	35.01	30.69			
Avera	31.936667	31.773333	31	33.34	35.93667	31.59			
Eq. lin	-1.00262	-0.953245	-0.72	-1.427	-2.21181	-0.89782			
RQ	0.0993986	0.1113667	0.19	0.0374	0.00614	0.126525			

Genes Normalised RQs (combined ref genes)							
	PBS 1	FW 1	PBS 2	FW 2	PBS 3	FW 3	
GroEL RQ	0.04240831	0.204304	0.02969996	0.15006297	0.69558306	0.40998666	
rpsJ/gyrA	0.10264	0.375424	0.012182	0.284769	0.272709	0.360145	
GroEL NR	0.413175	0.544195	2.438066	0.526965	2.550646	1.138395	
GroEL CN	1	1.3171058	1	0.21614038	1	0.44631623	0.659854

GroES RQ	0.26018033	0.8137691	0.07267354	0.74937926	2.14360906	1.95152404	
rpsJ/gyrA	0.10264	0.375424	0.012182	0.284769	0.272709	0.360145	
GroES NR	2.534879	2.167598	5.965761	2.631537	7.860437	5.418723	
GroES CN	1	0.85510904	1	0.44110675	1	0.68936666	0.661861

fliC RQ	0.99335957	0.61759271	0.40679443	0.47945567	1.17339557	0.59207475	
rpsJ/gyrA	0.10264	0.375424	0.012182	0.284769	0.272709	0.360145	
fliC NRQ	9.678082	1.645052	33.3937	1.683667	4.302745	1.643992	
fliC CNRQ	1	0.16997711	1	0.05041872	1	0.38207978	0.200825

fliD RQ	0.64606226	0.45144211	0.18302164	0.3782151	0.61224257	0.37991353	
rpsJ/gyrA	0.10264	0.375424	0.012182	0.284769	0.272709	0.360145	
fliD NRQ	6.294442	1.202485	15.02422	1.328149	2.245043	1.054892	
fliD CNRQ	1	0.19103919	1	0.08840051	1	0.46987592	0.249772

tcdA RQ	0.16440527	0.02690798	0.08688617	0.03768473	0.14760625	0.04717244	
rpsJ/gyrA	0.10264	0.375424	0.012182	0.284769	0.272709	0.360145	
tcdA NRQ	1.601764	0.071674	7.132474	0.132335	0.54126	0.130982	
tcdA CNR	1	0.0447466	1	0.01855381	1	0.24199452	0.101765

SpoVB RQ	0.0374245	0.09939856	0.00614031	0.1113667	0.12652506	0.19033751	
rpsJ/gyrA	0.10264	0.375424	0.012182	0.284769	0.272709	0.360145	
SpoVB NR	0.364619	0.264763	0.504057	0.391078	0.463957	0.528503	
SpoVB CN	1	0.72613751	1	0.7758601	1	1.13912083	0.880373

SpoIIIAC F	0.0006555	0.72189328	0.00091389	0.9704309	0.00561739	1.0172994	
rpsJ/gyrA	0.10264	0.375424	0.012182	0.284769	0.272709	0.360145	
SpoIIIAC N	0.006386	1.922873	0.075021	3.407787	0.020598	2.824697	
SpoIIIAC C	1	301.088666	1	45.4243811	1	137.131213	161.2148

Cspc RQ	0.01339265	3.18070149	0.00613869	2.87238883	0.06636735	2.58168961	
rpsJ/gyrA	0.10264	0.375424	0.012182	0.284769	0.272709	0.360145	
Cspc NRQ	0.130482	8.472284	0.503924	10.08675	0.243364	7.16848	
Cspc CNR	1	64.9308728	1	20.0164095	1	29.4558455	38.13438

Gene	ABS FC-seq	Abs FC PCR	RNA-Se	q-RT-PCI	fw/bhi rati	log2 (ratio)	FC (se	fw/bhi r	log2(ratic	FC (PCR)
groEL	1.235	-1.515486			1.235027	0.304542	1.24	0.6599	-0.5998	1.5154864
groES	1.24	-1.510892			1.243088	0.313928	1.24	0.6619	-0.5954	1.5108917
fliC	-4.44	-4.979455			0.22499	-2.15207	4.44	0.2008	-2.316	4.9794547
fliD	-3.25	-4.003653			0.307471	-1.70148	3.25	0.2498	-2.0013	4.0036534
tcdA	-4.01	-9.826564			0.249312	-2.00398	4.01	0.1018	-3.2967	9.8265635
SpoVI	1.52	1.1358824			1.528109	0.611748	1.53	0.8804	-0.1838	1.1358824
spoIII/	53	161.215			53.58596	5.743783	53.6	161.22	7.33284	161.215
cspC	2.27	38.1344			2.277007	1.187138	2.28	38.134	5.25302	38.1344

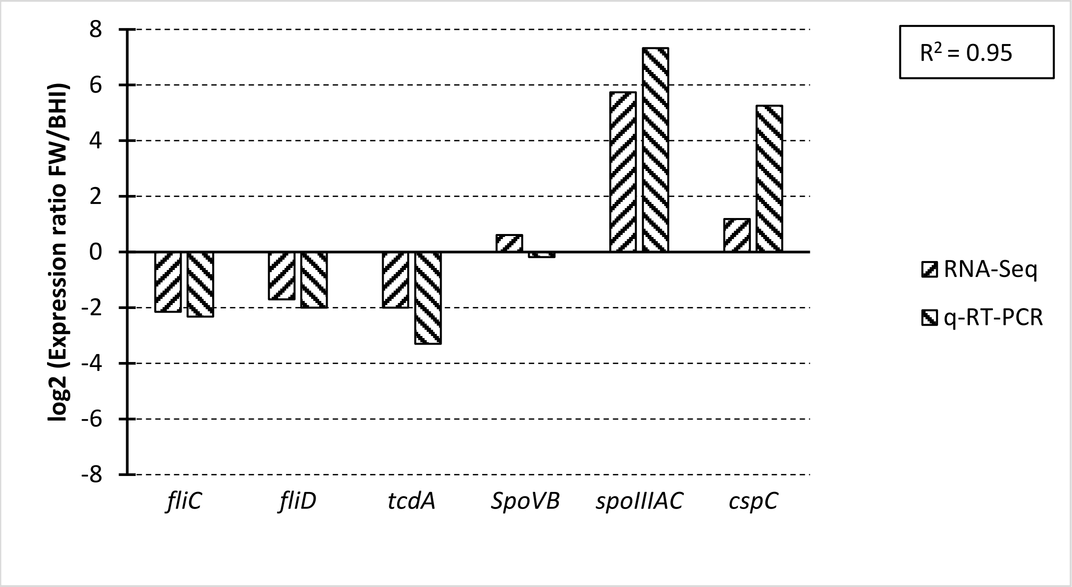

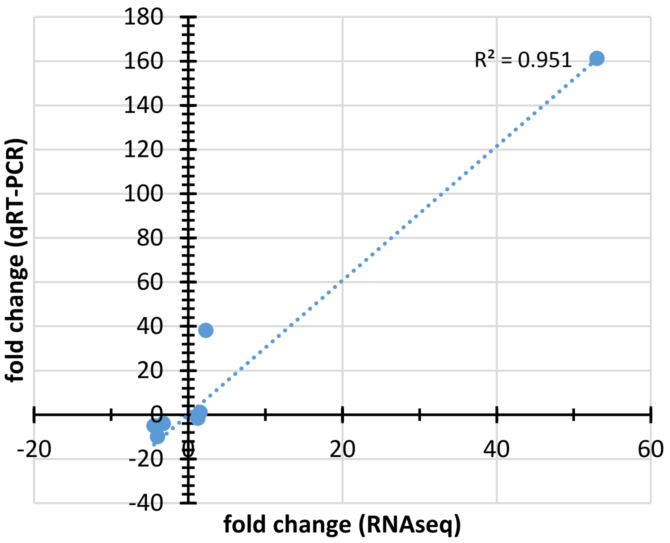

Var ID (Primary)	Var ID (Peak number)	Var ID (m/z)	Var ID (RT)	Var ID (new RT)	M3.pq[1]£
ID from XCMS	Peak number from XCMS	*m/z* from XCMS	RT from XCMS (secs)	RT (min)	OPLS loading value for start/end separation ¥
Peak_84	84	163.06	437.23	7.29	0.0472516
Peak_76	76	159.084	597.09	9.95	0.0470622
Peak_155	155	181.049	637.372	10.62	0.0468213
Peak_66	66	158.081	597.09	9.95	0.0462482
Peak_402	402	229.162	1766.39	29.44	0.0459237
Peak_526	526	247.118	1599.23	26.65	0.0458306
Peak_693	693	269.937	1476.94	24.62	0.0457825
Peak_42	42	149.027	429.581	7.16	0.0457636
Peak_241	241	200.128	1492.55	24.88	0.0456866
Peak_392	392	228.158	1764.9	29.42	0.0456618
Peak_517	517	246.115	1599.2	26.65	0.0456466
Peak_949	949	301.153	1823.83	30.40	0.045639
Peak_625	625	260.15	1600.93	26.68	0.0455534
Peak_733	733	274.163	1765.66	29.43	0.0454362
Peak_43	43	149.027	481.858	8.03	0.0454141
Peak_315	315	215.147	1600.19	26.67	0.0453767
Peak_2554	2554	492.232	1048.41	17.47	0.0452871
Peak_161	161	182.053	638.42	10.64	0.0452169
Peak_306	306	214.143	1600.19	26.67	0.0452106
Peak_46	46	151.023	481.927	8.03	0.0452027
Peak_498	498	244.117	1068.24	17.80	0.0450375
Peak_220	220	195.032	482.355	8.04	0.0449567
Peak_12	12	133.032	896.419	14.94	0.0448226
Peak_760	760	278.137	1463.31	24.39	0.0448189
Peak_770	770	279.14	1463.31	24.39	0.0447821
Peak_45	45	150.03	481.975	8.03	0.0447636
Peak_431	431	234.043	423.888	7.06	0.0447556
Peak_2095	2095	441.135	635.458	10.59	0.044482
Peak_529	529	248.127	1649.44	27.49	0.0441653
Peak_397	397	229.114	816.786	13.61	0.0440534
Peak_584	584	255.976	388.102	6.47	0.0438858
Peak_1023	1023	312.021	532.815	8.88	0.0438449
Peak_925	925	299.061	480.74	8.01	0.0438193
Peak_775	775	280.099	1651.94	27.53	0.0437381
Peak_375	375	226.143	1736.37	28.94	0.043731
Peak_2661	2661	505.218	962.273	16.04	0.0435085
Peak_641	641	262.143	1835.97	30.60	0.0433763
Peak_538	538	249.131	1650.32	27.51	0.0433653
Peak_648	648	263.146	1835.92	30.60	0.0433192

Peak_2259	2259	457.326	1765.17	29.42	0.0432816
Peak_33	33	144.066	310.862	5.18	0.0431098
Peak_295	295	212.127	1555.62	25.93	0.0428806
Peak_2004	2004	429.295	1601.28	26.69	0.0427017
Peak_1248	1248	337.166	442.26	7.37	0.0426582
Peak_186	186	187.116	1155.21	19.25	0.042639
Peak_2010	2010	430.298	1601.34	26.69	0.0425493
Peak_670	670	266.014	791.185	13.19	0.0423561
Peak_180	180	186.112	1156.05	19.27	0.042197
Peak_899	899	296.127	1875.42	31.26	0.0420681
Peak_657	657	264.122	1173.42	19.56	0.0420355
Peak_2301	2301	461.264	1600.3	26.67	0.0419657
Peak_148	148	179.07	1333.51	22.23	0.0419493
Peak_356	356	223.079	690.532	11.51	0.0417904
Peak_1678	1678	393.171	811.608	13.53	0.0417442
Peak_1527	1527	374.199	809.39	13.49	0.0417231
Peak_117	117	172.097	890.641	14.84	0.0416426
Peak_784	784	282.131	1600.19	26.67	0.0415138
Peak_1776	1776	404.185	801.794	13.36	0.0413602
Peak_349	349	222.076	690.49	11.51	0.0412013
Peak_659	659	264.135	1768.15	29.47	0.0411707
Peak_557	557	251.999	715.425	11.92	0.0410357
Peak_749	749	277.03	643.114	10.72	0.0409948
Peak_912	912	297.986	1073.52	17.89	0.0409513
Peak_1516	1516	373.194	809.377	13.49	0.0409468
Peak_1993	1993	429.141	436.553	7.28	0.0409405
Peak_2872	2872	535.068	659.626	10.99	0.040888
Peak_1027	1027	312.122	1569.9	26.17	0.0408584
Peak_72	72	159.065	1295.7	21.60	0.0408541
Peak_831	831	287.138	1662.57	27.71	0.0405759
Peak_1322	1322	347.161	1826.63	30.44	0.0404477
Peak_1779	1779	404.99	517.393	8.62	0.0404038
Peak_711	711	271.176	773.715	12.90	0.040304
Peak_9	9	131.07	807.093	13.45	0.0402816
Peak_3043	3043	557.283	1464.15	24.40	0.0402362
Peak_13	13	133.075	807.15	13.45	0.0402316
Peak_10	10	132.074	807.216	13.45	0.0401749
Peak_835	835	287.948	773.45	12.89	0.0398419
Peak_2657	2657	505.058	659.403	10.99	0.0398068
Peak_901	901	296.146	1765.23	29.42	0.0396868
Peak_40	40	147.044	998.911	16.65	0.0396767
Peak_1160	1160	327.996	1054.77	17.58	0.0396647
Peak_1736	1736	400.028	466.793	7.78	0.03962
Peak_1517	1517	373.232	1156.52	19.28	0.0395648
Peak_782	782	282.009	702.063	11.70	0.039292
Peak_75	75	159.102	1570.43	26.17	0.0392203
Peak_896	896	295.971	379.051	6.32	0.0391908
Peak_89	89	165.055	998.089	16.63	0.0388627

Peak_144	144	177.076	807.844	13.46	0.0388518
Peak_1241	1241	336.164	442.819	7.38	0.03877
Peak_1159	1159	327.996	1105.1	18.42	0.0387421
Peak_31	31	143.071	1094.9	18.25	0.0387302
Peak_299	299	213.086	354.038	5.90	0.0385932
Peak_94	94	166.058	998.231	16.64	0.0384828
Peak_44	44	149.06	1539.11	25.65	0.0384564
Peak_323	323	217.089	487.522	8.13	0.0384379
Peak_1621	1621	386.055	635.528	10.59	0.0383629
Peak_911	911	297.966	378.6	6.31	0.0383551
Peak_2630	2630	502.085	375.001	6.25	0.038315
Peak_542	542	250.053	1888.8	31.48	0.0382811
Peak_756	756	277.139	437.72	7.30	0.038262
Peak_2043	2043	435.148	783.484	13.06	0.038026
Peak_2656	2656	505.058	701.906	11.70	0.0379204
Peak_287	287	211.06	999.555	16.66	0.0378258
Peak_37	37	145.086	1207.15	20.12	0.0376526
Peak_38	38	146.089	1207.48	20.12	0.0372936
Peak_1796	1796	406.95	887.275	14.79	0.0368299
Peak_1491	1491	371.001	806.08	13.43	0.03661
Peak_3265	3265	592.437	1837.1	30.62	0.0365983
Peak_649	649	263.148	821.146	13.69	0.0364885
Peak_1895	1895	417.042	621.599	10.36	0.0364594
Peak_2702	2702	511.257	1010.96	16.85	0.0364194
Peak_188	188	187.116	1045.42	17.42	0.0362767
Peak_2135	2135	445.038	817.649	13.63	0.0362296
Peak_1611	1611	385.017	807.581	13.46	0.0362156
Peak_2338	2338	466.226	977.815	16.30	0.0360887
Peak_209	209	191.091	1209.16	20.15	0.0360786
Peak_179	179	186.112	1045.35	17.42	0.0360723
Peak_897	897	296.025	782.643	13.04	0.0359585
Peak_661	661	264.151	807.129	13.45	0.0359144
Peak_3610	3610	644.36	1155.03	19.25	0.0358939
Peak_2134	2134	445.038	780.782	13.01	0.0358924
Peak_317	317	216.032	587.071	9.78	0.0358896
Peak_67	67	158.081	532.159	8.87	0.0358353
Peak_1610	1610	385.017	855.01	14.25	0.0357029
Peak_1141	1141	326.034	786.235	13.10	0.0356851
Peak_2695	2695	510.252	1009.75	16.83	0.0356757
Peak_3423	3423	615.248	937.481	15.62	0.0355905
Peak_592	592	257.113	472.271	7.87	0.0355377
Peak_1246	1246	337.088	854.838	14.25	0.0354925
Peak_2253	2253	457.189	809.601	13.49	0.0354584
Peak_2708	2708	512.3	958.502	15.98	0.0352402
Peak_682	682	268.08	690.183	11.50	0.0352094
Peak_1747	1747	401.011	775.498	12.92	0.0351946
Peak_898	898	296.025	847.189	14.12	0.0351271
Peak_664	664	265.081	391.378	6.52	0.0350675

Peak_1388	1388	357.023	342.794	5.71	0.0350508
Peak_2368	2368	469.209	572.549	9.54	0.0349285
Peak_1875	1875	415.027	792.332	13.21	0.034904
Peak_553	553	251.11	1005.12	16.75	0.0348259
Peak_2460	2460	482.222	716.656	11.94	0.0347039
Peak_2355	2355	468.205	640.879	10.68	0.0346821
Peak_543	543	250.107	1004.78	16.75	0.0346478
Peak_251	251	201.131	1327.06	22.12	0.0346218
Peak_3418	3418	614.246	937.481	15.62	0.0344583
Peak_830	830	287.138	1703.83	28.40	0.0344366
Peak_2190	2190	450.186	691.687	11.53	0.0344327
Peak_507	507	245.091	1198.79	19.98	0.0341103
Peak_308	308	215.032	435.245	7.25	0.0339688
Peak_240	240	200.128	1326.3	22.11	0.0339647
Peak_1835	1835	411.185	653.419	10.89	0.0339537
Peak_1693	1693	395.143	429.431	7.16	0.033872
Peak_992	992	307.092	490.799	8.18	0.0338399
Peak_521	521	246.133	1389.57	23.16	0.0336581
Peak_2291	2291	461.032	613.641	10.23	0.0336137
Peak_725	725	273.122	1537.67	25.63	0.0335035
Peak_1530	1530	374.979	652.287	10.87	0.0334123
Peak_489	489	243.13	894.282	14.90	0.033404
Peak_1872	1872	414.232	850.831	14.18	0.0333774
Peak_2354	2354	468.205	572.471	9.54	0.0333714
Peak_583	583	255.922	1171.31	19.52	0.033286
Peak_545	545	250.12	1600.02	26.67	0.033277
Peak_593	593	257.113	404.448	6.74	0.0332483
Peak_3371	3371	608.229	995.56	16.59	0.0332244
Peak_1871	1871	414.208	896.247	14.94	0.0331739
Peak_587	587	256.19	2076.42	34.61	0.0331665
Peak_3150	3150	574.219	590.808	9.85	0.0331077
Peak_1877	1877	415.026	841.133	14.02	0.0329687
Peak_2582	2582	496.238	875.326	14.59	0.0327649
Peak_2387	2387	471.278	689.354	11.49	0.032702
Peak_1142	1142	326.034	723.273	12.05	0.032681
Peak_2943	2943	544.237	1181.33	19.69	0.0326796
Peak_139	139	175.061	625.271	10.42	0.0326587
Peak_590	590	257.113	562.57	9.38	0.0326504
Peak_846	846	289.03	583.73	9.73	0.032612
Peak_1876	1876	415.026	884.225	14.74	0.0326002
Peak_504	504	245.011	653.666	10.89	0.0325121
Peak_2706	2706	512.231	810.849	13.51	0.0325051
Peak_502	502	244.173	690.805	11.51	0.032328
Peak_3022	3022	555.167	424.69	7.08	0.0321186
Peak_319	319	216.086	486.445	8.11	0.0321175
Peak_242	242	200.128	1378.38	22.97	0.0321009
Peak_252	252	201.131	1378.38	22.97	0.0320888
Peak_3102	3102	568.294	897.282	14.95	0.0320466

Peak_2136	2136	445.038	736.887	12.28	0.0319163
Peak_329	329	218.102	970.174	16.17	0.0316832
Peak_591	591	257.113	363.535	6.06	0.0316365
Peak_1709	1709	397.072	681.581	11.36	0.0316107
Peak_490	490	243.17	692.119	11.54	0.0315971
Peak_658	658	264.122	1240.3	20.67	0.0315761
Peak_1874	1874	414.222	977.682	16.29	0.0315201
Peak_2318	2318	464.172	607.505	10.13	0.0315022
Peak_3782	3782	671.279	1494	24.90	0.031277
Peak_2694	2694	510.252	938.838	15.65	0.0312445
Peak_876	876	292.153	1669.1	27.82	0.031233
Peak_1272	1272	341.178	621.518	10.36	0.0312006
Peak_267	267	204.066	1149.15	19.15	0.031191
Peak_735	735	275.069	386.447	6.44	0.031146
Peak_495	495	244.027	634.543	10.58	0.0311056
Peak_1494	1494	371.04	492.238	8.20	0.0306576
Peak_3730	3730	664.327	1010.51	16.84	0.0306343
Peak_286	286	211.032	620.987	10.35	0.03057
Peak_2715	2715	513.244	810.549	13.51	0.0305576
Peak_3956	3956	710.364	907.286	15.12	0.0303844
Peak_845	845	289.03	663.951	11.07	0.0303685
Peak_1158	1158	327.942	714.194	11.90	0.0303639
Peak_1466	1466	367.194	803.156	13.39	0.0303125
Peak_1624	1624	386.156	639.612	10.66	0.0303042
Peak_1091	1091	317.182	529.042	8.82	0.0302528
Peak_1492	1492	371.001	747.876	12.46	0.0302022
Peak_2170	2170	448.099	498.185	8.30	0.030195
Peak_326	326	218.012	369.144	6.15	0.0301734
Peak_860	860	290.033	659.641	10.99	0.0301247
Peak_3757	3757	668.283	1462.27	24.37	0.0299048
Peak_1147	1147	326.206	1709.43	28.49	0.0298698
Peak_234	234	199.071	700.238	11.67	0.0298681
Peak_2621	2621	501.124	309.44	5.16	0.029824
Peak_2590	2590	497.243	802.277	13.37	0.0297917
Peak_1236	1236	336.117	342.802	5.71	0.0297766
Peak_2581	2581	496.238	802.04	13.37	0.0297385
Peak_3888	3888	695.413	1681.32	28.02	0.029728
Peak_298	298	213.086	433.541	7.23	0.0297034
Peak_1914	1914	418.967	517.331	8.62	0.0296758
Peak_861	861	290.113	342.792	5.71	0.0296743
Peak_305	305	214.09	433.541	7.23	0.0296284
Peak_603	603	258.115	404.32	6.74	0.0295945
Peak_1529	1529	374.979	579.913	9.67	0.0295187
Peak_1623	1623	386.174	598.898	9.98	0.0294769
Peak_3209	3209	585.225	346.562	5.78	0.0294591
Peak_1112	1112	320.933	328.842	5.48	0.0293693
Peak_374	374	226.118	567.368	9.46	0.0293329
Peak_1247	1247	337.12	342.792	5.71	0.0293198

Peak_1979	1979	427.215	969.461	16.16	0.029252
Peak_1139	1139	325.138	1838.29	30.64	0.0292214
Peak_1124	1124	322.928	327.33	5.46	0.02921
Peak_1920	1920	420.104	474.481	7.91	0.0291679
Peak_1472	1472	368.198	803.456	13.39	0.0290473
Peak_2459	2459	482.222	778.198	12.97	0.0288502
Peak_1282	1282	342.164	794.333	13.24	0.0288243
Peak_2545	2545	491.174	428.64	7.14	0.0286792
Peak_2367	2367	469.208	642.159	10.70	0.0286193
Peak_1762	1762	402.197	500.12	8.34	0.0286153
Peak_3883	3883	694.299	962.903	16.05	0.028566
Peak_2120	2120	443.21	870.528	14.51	0.0285206
Peak_339	339	220.026	374.204	6.24	0.0285085
Peak_630	630	261.054	358.617	5.98	0.0284629
Peak_1861	1861	413.196	1066.44	17.77	0.0284231
Peak_535	535	249.086	306.415	5.11	0.0283767
Peak_2927	2927	542.217	798.204	13.30	0.0282413
Peak_118	118	172.097	788.771	13.15	0.0282325
Peak_3952	3952	710.23	375.329	6.26	0.0281734
Peak_1873	1873	414.201	1330.69	22.18	0.0281639
Peak_1757	1757	401.262	1388.79	23.15	0.0280173
Peak_1740	1740	400.168	937.099	15.62	0.0279857
Peak_4257	4257	777.339	828.078	13.80	0.0279767
Peak_931	931	299.159	1003.21	16.72	0.0279215
Peak_2521	2521	487.347	692.738	11.55	0.0279081
Peak_2306	2306	462.221	1042.98	17.38	0.027887
Peak_4067	4067	730.332	961.75	16.03	0.0278281
Peak_2232	2232	455.212	669.758	11.16	0.0278119
Peak_2031	2031	433.199	341.565	5.69	0.0277859
Peak_2560	2560	493.204	1044.43	17.41	0.0277503
Peak_1928	1928	421.103	477.434	7.96	0.0277418
Peak_3645	3645	651.342	890.106	14.84	0.0277416
Peak_3827	3827	680.25	1041.19	17.35	0.0276799
Peak_935	935	300.065	477.031	7.95	0.0276048
Peak_1368	1368	355.042	598.55	9.98	0.0275815
Peak_1607	1607	384.21	1277.17	21.29	0.027536
Peak_4557	4557	896.483	1792.28	29.87	0.0275275
Peak_3567	3567	638.274	678.955	11.32	0.027416
Peak_3481	3481	624.391	1154.56	19.24	0.0274112
Peak_271	271	206.081	1076	17.93	0.0273957
Peak_1778	1778	404.99	598.177	9.97	0.0273663
Peak_866	866	291.096	879.011	14.65	0.0273615
Peak_2161	2161	447.185	1071.67	17.86	0.0272893
Peak_413	413	231.014	483.646	8.06	0.0272776
Peak_273	273	207.084	1076.02	17.93	0.0272583
Peak_2377	2377	470.221	819.503	13.66	0.0272496
Peak_3611	3611	645.177	368.054	6.13	0.027221
Peak_3988	3988	714.324	1006.53	16.78	0.0272162

Peak_536	536	249.086	603.641	10.06	0.0272151
Peak_678	678	267.092	430.842	7.18	0.0272089
Peak_750	750	277.081	423.665	7.06	0.0271614
Peak_1601	1601	384.059	646.737	10.78	0.0270683
Peak_1857	1857	413.114	1778.39	29.64	0.0270665
Peak_2934	2934	543.226	797.392	13.29	0.0269677
Peak_2707	2707	512.231	853.945	14.23	0.02695
Peak_2507	2507	486.253	848.955	14.15	0.0269481
Peak_905	905	297.042	839.253	13.99	0.0269308
Peak_915	915	298.105	1392.05	23.20	0.0269211
Peak_4379	4379	811.513	1810.12	30.17	0.0268904
Peak_758	758	278.084	423.435	7.06	0.0268388
Peak_4553	4553	895.481	1796.54	29.94	0.026801
Peak_4169	4169	753.298	574.845	9.58	0.026752
Peak_262	262	203.081	604.533	10.08	0.0266827
Peak_483	483	242.157	1927.8	32.13	0.0265721
Peak_1956	1956	424.09	707.025	11.78	0.0264982
Peak_1042	1042	313.174	1200.54	20.01	0.0264518
Peak_2275	2275	459.205	756.394	12.61	0.0263973
Peak_991	991	307.092	566.344	9.44	0.0263924
Peak_1855	1855	413.065	597.001	9.95	0.0263713
Peak_2252	2252	457.189	757.196	12.62	0.0262935
Peak_49	49	151.039	802.872	13.38	0.0262885
Peak_1040	1040	313.157	782.753	13.05	0.0262872
Peak_1862	1862	413.202	894.022	14.90	0.026199
Peak_2568	2568	495.114	367.594	6.13	0.026176
Peak_2346	2346	467.185	329.65	5.49	0.026116
Peak_2413	2413	475.047	660.879	11.01	0.0260992
Peak_3923	3923	702.274	319.336	5.32	0.0260842
Peak_1003	1003	309.073	594.36	9.91	0.026034
Peak_1593	1593	383.055	645.854	10.76	0.026017
Peak_1782	1782	405.138	750.279	12.50	0.0259241
Peak_3565	3565	637.446	1768.34	29.47	0.0259024
Peak_1833	1833	411.185	606.049	10.10	0.0258795
Peak_1354	1354	353.046	598.55	9.98	0.0258761
Peak_3558	3558	636.444	1767.99	29.47	0.0258641
Peak_1866	1866	414.069	598.375	9.97	0.0258148
Peak_1803	1803	407.199	447.013	7.45	0.0258137
Peak_2950	2950	545.125	745.318	12.42	0.0257632
Peak_1132	1132	323.538	548.305	9.14	0.0256843
Peak_1986	1986	428.174	367.186	6.12	0.0256842
Peak_2479	2479	484.048	780.154	13.00	0.0256842
Peak_1532	1532	375.066	623.38	10.39	0.0256669
Peak_2113	2113	443.076	552.651	9.21	0.0256629
Peak_1651	1651	389.201	519.334	8.66	0.0256551
Peak_2373	2373	470.156	905.969	15.10	0.0255939
Peak_1210	1210	332.144	350.959	5.85	0.0255631
Peak_1969	1969	426.16	374.677	6.24	0.0255354

Peak_1867	1867	414.069	542.716	9.05	0.0255119
Peak_3970	3970	712.34	1018.34	16.97	0.0254196
Peak_3352	3352	604.312	1821.41	30.36	0.0253994
Peak_4477	4477	854.332	373.957	6.23	0.0253929
Peak_3615	3615	646.181	372.689	6.21	0.0253698
Peak_3112	3112	570.249	577.524	9.63	0.0253684
Peak_2391	2391	472.279	689.584	11.49	0.0253212
Peak_1613	1613	385.051	648.161	10.80	0.025268
Peak_609	609	259.023	1542.51	25.71	0.0251842
Peak_530	530	248.901	751.638	12.53	0.0251672
Peak_3521	3521	629.274	413.979	6.90	0.0251003
Peak_2489	2489	485.184	803.747	13.40	0.0250848
Peak_786	786	283.066	375.645	6.26	0.0250609
Peak_2027	2027	433.169	927.507	15.46	0.0250498
Peak_254	254	202.016	460.914	7.68	0.025049
Peak_1351	1351	352.904	585.873	9.76	0.0250197

£Titles from SIMCA
¥Explanation
